# Assessing transcriptomic heterogeneity of single-cell RNASeq data by bulk-level gene expression data

**DOI:** 10.1186/s12859-024-05825-3

**Published:** 2024-06-12

**Authors:** Khong-Loon Tiong, Dmytro Luzhbin, Chen-Hsiang Yeang

**Affiliations:** Institute of Statistical Science, Academia Sinica, Taipei, Taiwan

**Keywords:** Single-cell RNASeq data, Deconvolution, Probabilistic graphical models, Heterogeneity

## Abstract

**Background:**

Single-cell RNA sequencing (sc-RNASeq) data illuminate transcriptomic heterogeneity but also possess a high level of noise, abundant missing entries and sometimes inadequate or no cell type annotations at all. Bulk-level gene expression data lack direct information of cell population composition but are more robust and complete and often better annotated. We propose a modeling framework to integrate bulk-level and single-cell RNASeq data to address the deficiencies and leverage the mutual strengths of each type of data and enable a more comprehensive inference of their transcriptomic heterogeneity. Contrary to the standard approaches of factorizing the bulk-level data with one algorithm and (for some methods) treating single-cell RNASeq data as references to decompose bulk-level data, we employed multiple deconvolution algorithms to factorize the bulk-level data, constructed the probabilistic graphical models of cell-level gene expressions from the decomposition outcomes, and compared the log-likelihood scores of these models in single-cell data. We term this framework *backward deconvolution* as inference operates from coarse-grained bulk-level data to fine-grained single-cell data. As the abundant missing entries in sc-RNASeq data have a significant effect on log-likelihood scores, we also developed a criterion for inclusion or exclusion of zero entries in log-likelihood score computation.

**Results:**

We selected nine deconvolution algorithms and validated backward deconvolution in five datasets. In the in-silico mixtures of mouse sc-RNASeq data, the log-likelihood scores of the deconvolution algorithms were strongly anticorrelated with their errors of mixture coefficients and cell type specific gene expression signatures. In the true bulk-level mouse data, the sample mixture coefficients were unknown but the log-likelihood scores were strongly correlated with accuracy rates of inferred cell types. In the data of autism spectrum disorder (ASD) and normal controls, we found that ASD brains possessed higher fractions of astrocytes and lower fractions of NRGN-expressing neurons than normal controls. In datasets of breast cancer and low-grade gliomas (LGG), we compared the log-likelihood scores of three simple hypotheses about the gene expression patterns of the cell types underlying the tumor subtypes. The model that tumors of each subtype were dominated by one cell type persistently outperformed an alternative model that each cell type had elevated expression in one gene group and tumors were mixtures of those cell types. Superiority of the former model is also supported by comparing the real breast cancer sc-RNASeq clusters with those generated by simulated sc-RNASeq data.

**Conclusions:**

The results indicate that backward deconvolution serves as a sensible model selection tool for deconvolution algorithms and facilitates discerning hypotheses about cell type compositions underlying heterogeneous specimens such as tumors.

**Supplementary Information:**

The online version contains supplementary material available at 10.1186/s12859-024-05825-3.

## Introduction

Transcriptomic heterogeneity is probed by RNA sequencing data at bulk and single-cell levels. Each type of data has its merits and shortcomings. Bulk-level RNASeq data fail to directly disclose subpopulation variability in a heterogeneous sample, but are less vulnerable to measurement noise and missing values and often better annotated. Single-cell RNASeq (sc-RNASeq) data directly manifest cellular heterogeneity, but also suffer from high measurement noise and dropouts, and sometimes lack proper annotations. Integrating bulk-level and single-cell RNASeq data by leveraging their complementary merits can address these deficiencies and provide a more comprehensive understanding of the heterogeneous cell types and their compositions in samples.

A standard approach for unveiling heterogeneity from bulk-level data is *deconvolution*. Denote an $n\times m$ matrix $E$ the expression data of $n$ genes and $m$ bulk samples. Assume each bulk sample comprises cells from $k$ types, and the expression data of a sample is the weighted sum of the expression profiles of the $k$ cell types. Under these assumptions $E$ is approximately factorized into the product of two matrices:

1 $$E \approx Q \cdot P.$$

$Q$ is an $n\times k$ *signature matrix* denoting the expression profiles of $n$ genes in $k$ cell types, and $P$ is a $k\times m$ *mixture coefficient matrix* denoting the proportions of $k$ cell types in $m$ samples, where the $P$ entries in each column are nonnegative and sum to 1, and the $Q$ entries are nonnegative as well.

Numerous deconvolution algorithms have been proposed (see reviews [1, 2]), and they fall into two general categories. Complete deconvolution methods simultaneously solve $Q$ and $P$ from $E$ by imposing various constraints on the inferred matrices [3, 4]. Incomplete deconvolution methods take one matrix either as given or inferred from external sources and optimize the other matrix. Very few incomplete deconvolution methods fix $P$ and optimize $Q$ [5], and the majority of the methods fix $Q$ and optimize $P$ [6–11]. $Q$ is either explicitly given as an input [12], constructed from cell type specific marker genes [13], derived from the reference expression profiles at bulk-level [7] or single-cell RNASeq data [14–18]. Several benchmark studies also extensively compared multiple deconvolution methods in a wide range of experimental datasets and performance settings [1, 19, 20].

Despite diversity and richness of these methods, integration of bulk-level and single-cell data (if undertaken) is solely achieved by utilizing the single-cell data as a reference to infer the composition of the bulk-level data.

Inference from single-cell to bulk-level data directly matches the goal of deconvolution as the signature matrix $Q$ can be directly derived from the single-cell data. Hence we term this inference direction forward deconvolution. However, forward deconvolution may be infeasible or misleading as single-cell data are sparse, noisy and sometimes unannotated. A prominent example is cancer transcriptomics data. Tumor subtypes have distinct expression patterns in their bulk-level RNASeq data [21–23]. Yet in the single-cell data the cancer cell types are often unannotated and the data quality is substantially inferior, as indicated in prior studies [24, 25] and our analysis on the data of breast cancer and low grade gliomas in the Results section. Therefore, prior deconvolution methods using single-cell RNASeq data as a reference are not applicable in certain contexts.

To fix these caveats of forward (single-cell $\to$ bulk-level data) deconvolution, we propose a *backward deconvolution* framework to integrate bulk-level and single-cell RNASeq data and simultaneously infer (1) signature expression profiles of cell types, (2) mixture coefficients of cell types in each bulk sample, (3) relations between expression profiles of bulk-level sample subtypes and cell types, (4) cell type assignments in single-cell RNASeq data. Backward deconvolution employs several forward deconvolution algorithms to the bulk-level data and derives the probabilistic graphical models of single-cell gene expressions from the deconvolution results. It then evaluates the log-likelihood scores of these models in the single-cell data and selects the best model according to its log-likelihood score.

Representation of sc-RNASeq data as a probabilistic graphical model has been proposed since the early stage of single-cell technology development. The most common approach is to borrow topic models or Latent Dirichlet Allocation (LDA) in text analysis [26] to the sc-RNASeq data. LDA models word distributions per topic and topic distributions per document with two nested Dirichlet distributions. There is a direct correspondence from documents, words and latent topics in text analysis to cells, genes and cell functions in sc-RNASeq data. LDA is now widely used in dimension reduction [27, 28] and clustering [29] of sc-RNASeq data alone. A more relevant approach unifies bulk-level and single-cell RNASeq data with a more general probabilistic graphical model (URSM [10]). Our work shares a common spirit of a hierarchical probabilistic representation of the data generation process but substantially differs from them in several important aspects. Most LDA studies on gene expression data apply to single-cell RNASeq data only and fail to integrate both single-cell and bulk-level data. Although URSM tackles integration of both types of data, the graphical model is based on one set of particular assumptions about the data. In contrast, backward deconvolution directly tackles bulk-level and single-cell data integration and allows multiple modeling assumptions encoded by different forward deconvolution algorithms. These features are unique in our approach.

We justified backward deconvolution by selecting nine deconvolution algorithms and applying the framework to five single-cell and bulk-level datasets: (1) the sc-RNASeq data of mouse gene expressions and its in-silico mixtures as the virtual bulk-level data, (2) the true bulk-level and single-cell RNASeq data of mouse gene expressions, (3) the bulk-level and single-cell RNASeq data of the brains of ASD patients and normal controls, (4) the breast cancer bulk-level and single-cell RNASeq data, (5) the low-grade gliomas bulk-level and single-cell RNASeq data. In the mouse datasets with cell type annotations, the log-likelihood scores were aligned with several common performance metrics such as the accuracy rate of predicted cell type assignments, similarity between true and inferred signature matrices, and similarity between true and inferred mixture coefficients matrices. In the ASD data, backward deconvolution outcomes indicated that ASD brains possessed higher fractions of astrocytes and lower fractions of NRGN-expressing neurons. In the cancer datasets with no cell type annotations, we compared three simple hypotheses about cell type expression patterns and found the model that cancer cells of each subtype were dominated by one cell type was superior to other models. The results indicate that backward deconvolution (1) is a sensible model selection tool for deconvolution algorithms and (2) facilitates discerning hypotheses about cell type compositions underlying heterogeneous specimens.

## Materials and methods

### Overview of the backward deconvolution framework

The objective of backward deconvolution is to simultaneously infer the gene expression signatures of the underlying cell types and their compositions in selected sample types from both bulk-level and single-cell RNASeq data. The outcome of a standard deconvolution algorithm (forward deconvolution) is a decomposition of bulk-level data as in Eq. 1. Our method converts the decomposition outcome into a probabilistic graphical model for single-cell gene expressions, and applies the model to fit single-cell data. We term this method *backward deconvolution* as inference is undertaken from bulk-level to single-cell data. Rather than incurring one algorithm to perform decomposition, backward deconvolution compares several forward deconvolution algorithms on their goodness of fit to the single-cell data and selects the best one. Therefore, it should be viewed as a framework of constructing and selecting models from multiple deconvolution algorithms.

**Fig. 1:**
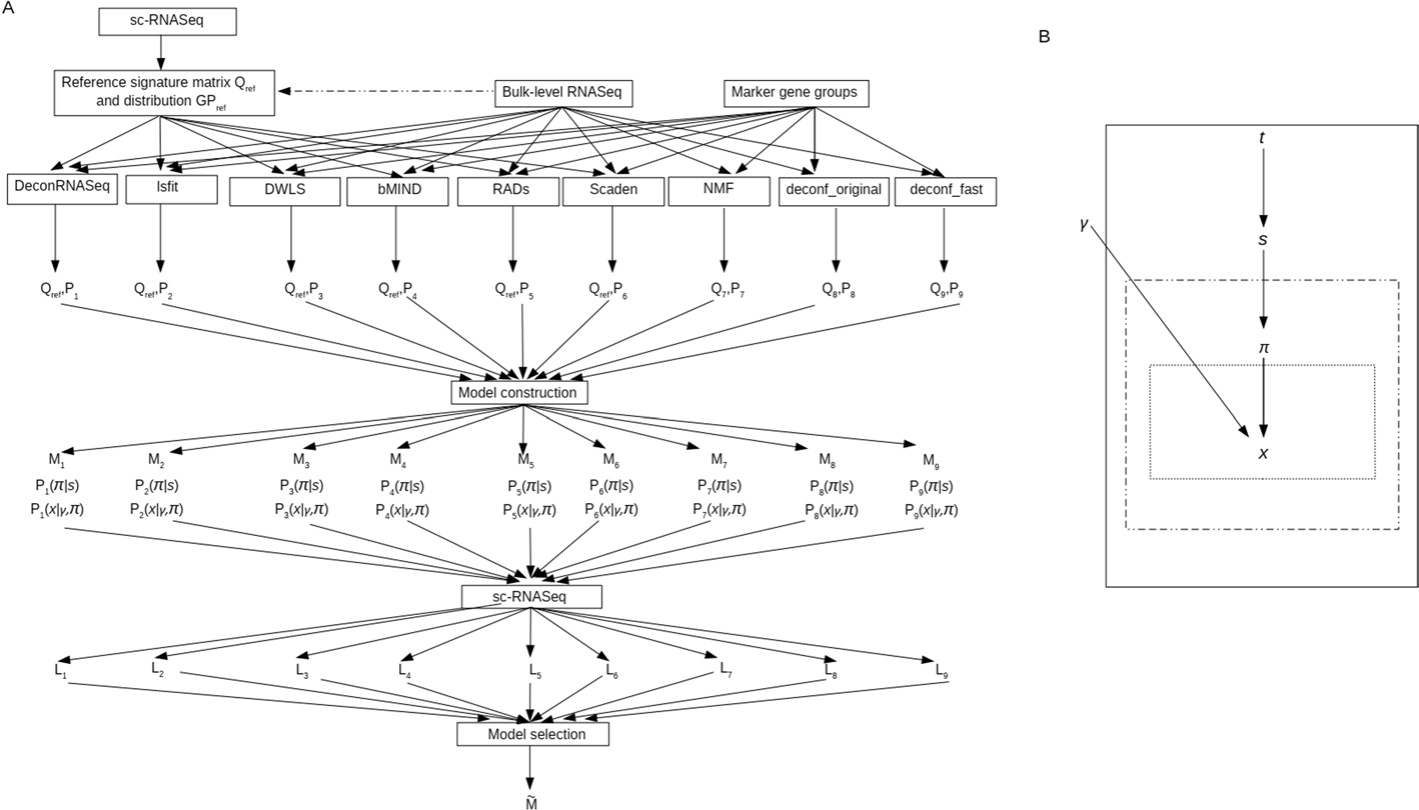
**A** Overview of the backward deconvolution framework. Single-cell and bulk-level RNASeq data are used to generate reference signature matrix and distribution. Nine deconvolution algorithms decompose the bulk-level data into $Q \cdot P$. For six incomplete methods $Q_{ref}$ and $GP_{ref}$ are derived from the single-cell data. The deconvolution outcomes are converted into probabilistic graphical models. Finally, these models are employed to the sc-RNASeq data to evaluate log-likelihood scores. The model with the highest score is selected. **B** The probabilistic graphical model of cell-level gene expressions. It is represented by a plate notation. $t, s, \pi ,\gamma ,x$ denote bulk sample identity, sample subtype, cell type, gene group index, and gene expression respectively. The three boxes from outside in denote individual bulk samples, cells within each sample, and gene expressions within each cell. The terms pertaining to an interior box are repeated multiple times for each instance pertaining to an exterior box. Within each sample the term $P(\pi |s)$ is repeated for each cell. Within each cell the term $P(x|\gamma ,\pi )$ is repeated for each gene. The likelihood of the joint model is depicted in Eq. 2

Figure 1A illustrates the backward deconvolution framework. The inputs include (1) bulk-level RNASeq data of samples labeled with subtypes, (2) single-cell RNASeq data of samples from the same bulk-level subtypes (but not necessarily from the same specimens of the bulk-level RNASeq data), (3) a subset of marker genes pertaining to the sample subtypes, and group labels of the marker genes whose expression profiles distinguish sample subtypes, (4) a number of forward deconvolution algorithms. The outputs include (1) transcription signatures of gene markers for each cell type, (2) mixture coefficients of cell types in each bulk sample, (3) the probabilistic graphical model of the deconvolution algorithm that best fits the single-cell data, (4) cell type assignments in sc-RNASeq data. Briefly, it consists of the following steps. First, we generate a reference signature matrix and a reference signature distribution from single-cell or bulk-level RNASeq data. A reference signature matrix comprises the mean marker gene expressions of each cell type. A reference signature distribution specifies the expression distribution of each marker gene group in each cell type. Second, we employ multiple forward deconvolution algorithms to factorize the bulk-level data (Eq. 1). Third, from the outcome of each deconvolution algorithm we construct a probabilistic graphical model for single-cell gene expressions. Finally, we evaluate the marginal log-likelihood scores of all models in the single-cell data and select the best model based on the log-likelihood scores. In each model we also assign each cell to a cell type according to its posterior probabilities of cell types given the sc-RNASeq data.

### Fitting single-cell gene expression data with probabilistic graphical models constructed from forward deconvolution outcomes

The major highlight contrasting backward deconvolution with canonical forward deconvolution approaches is to employ the deconvolution outcomes of the bulk-level data to fit the single-cell gene expression data. Here we give a brief preview of this approach and provide details of each step in subsequent sections. We assume both single-cell and bulk-level RNASeq data are generated from the same process which can be represented by a probabilistic graphical model with a hierarchical structure. Samples in the data are drawn from several subtypes (e.g., different tissue types or cancer subtypes). Each sample constitutes cells belonging to several cell types where the cell type composition depends on the sample subtype. Cells of each type possess a specific expression signature of selected marker genes. Furthermore, the marker genes are categorized into several groups where members of each gene group possess similar expression patterns across cell types. Single-cell RNASeq data are noisy measurements of the marker gene expressions of individual representative cells from the process. Bulk-level RNASeq data are measurements of the marker gene expressions of mixtures of the representative cells.

More precisely, denote $t, s, \pi , \gamma , x$ random variables of sample identities, sample subtypes, cell types, gene group labels, and individual gene expressions, respectively. The probabilistic graphical model constitutes two families of parameters: $P\left(\pi |s\right)$’s specify the conditional probabilities of cell types given sample subtypes, and $P(x|$ $\gamma ,\pi )$’s specify the conditional probabilities of marker gene expressions given gene group labels and cell types. A complete model should also include prior probabilities $P(s)$ and $P(\gamma )$. These priors are discarded here as the sample subtypes are determined by the sample identities $t$ and the gene group labels are determined by gene identities. All these variables have subscript indices denoting individual genes, cells or bulk samples. The joint likelihood of observing the sc-RNASeq data becomes:

2 $$\mathcal{L}(T,S,\Pi ,X)=\prod_{i}P\left({s}_{i}|{t}_{i}\right)\prod_{j}P\left({\pi }_{ij}|{s}_{i}\right)\prod_{l}P({x}_{ijl}|{\gamma }_{l},{\pi }_{ij}).$$

where indices $i, j, l$ are over samples, cells and genes respectively. Evaluation of the joint likelihood resembles the sampling process and can be concisely represented by a plate notation illustrated in Fig. 1B. Multiplications of the terms over the three indices ($l, j, i$) are represented as nested boxes inside out. The term $P\left({s}_{i}|{t}_{i}\right)$ is deterministic as the subtype of each sample is unique and known.

Forward deconvolution can be viewed as inference of these model parameters from the bulk-level (and single-cell) RNASeq data. Complete methods infer $Q$ and $P$ from the bulk-level data, and we can derive $P(x|$ $\gamma ,\pi )$ and $P\left(\pi |s\right)$ accordingly. Incomplete methods infer $P$ from the bulk-level data and reference single-cell data, and we can derive $P\left(\pi |s\right)$ according to $P$ and directly construct $P(x|$ $\gamma ,\pi )$ from the reference single-cell data. Once these parameters are decided, we plug them into Eq. 2 and evaluate the likelihood score of the sc-RNASeq data.

In some applications cell type labels are unobserved. For instance, in tumor data typically normal cells are annotated but cancer cells are not since there are few standard ways to delineate cancer cell subtypes. To cope with this scenario we evaluate the marginal likelihood function over possible cell type labels:

3 $$\mathcal{L}(T,S,X)=\prod_{i}P\left({s}_{i}|{t}_{i}\right)\prod_{j}\sum_{{\pi }_{ij}}P\left({\pi }_{ij}|{s}_{i}\right)\prod_{l}P({x}_{ijl}|{\gamma }_{l},{\pi }_{ij}).$$

The log (marginal) likelihood score quantifies the goodness of fit of a deconvolution model to the sc-RNASeq data but does not take model complexity into account. We add a regularization term to the log-likelihood value and evaluate the Bayesian Information Criterion (BIC) score [30]:

4 $${\mathcal{L}}_{B}\left(T,S,X\right)=\text{log}\mathcal{L}(T,S,X)-\frac{D}{2}\text{log}N.$$

$N$ is the number of cells in the sc-RNASeq data and $D$ is the degree of freedom in the model. $D$ is determined by the number of independent entries in $P\left(\pi |s\right)$ and $P(x|$ $\gamma ,\pi )$.

The backward deconvolution framework applies several existing forward deconvolution algorithms to the bulk-level RNASeq data and uses the BIC scores (Eq. 4) to measure the goodness of the inferred parameters to fit the sc-RNASeq data. Despite the counterintuitive inference direction from bulk-level to single-cell data, this approach has several advantages. The framework can be treated as an ensemble learning method if we want to deconvolve bulk-level data by combining multiple forward deconvolution algorithms, or a model selection criterion if we want to compare the performance of multiple deconvolution methods. It also offers a more robust way to integrate both bulk-level and single-cell expression data since it allows multiple assumptions about the relations between bulk-level and single-cell data and can tolerate noisy and missing entries as well as lack of annotations in single-cell data.

### Generating reference signature matrices and distributions from the single-cell data

A reference signature matrix specifies the mean expression value of each marker gene in each cell type. A reference distribution specifies the expression distribution of each marker gene group in each cell type. Both are derived from single-cell or bulk-level data. In this section, we describe the procedures of deriving these quantities from the single-cell data if the sc-RNASeq data have cell type annotations and reliable quality.

For incomplete deconvolution algorithms, the reference signature matrix ${Q}_{ref}$ ($Q$ in Eq. 1) is directly obtained from the sc-RNASeq data. Denote $X$ an $n\times {N}_{c}$ matrix of sc-RNASeq data with $n$ genes and ${N}_{c}$ cells, and $\tau$ a $1\times {N}_{c}$ vector of $k$ cell type annotations of the ${N}_{c}$ cells. The reference signature matrix ${Q}_{ref}$ is an $n\times k$ matrix where ${Q}_{ref}\left(i,j\right)=\frac{1}{{N}_{j}}\sum_{\{l:\tau \left(l\right)=j\}}{X}_{il}$ is the average expression of gene $i$ over type $j$ cells in $X$ (${N}_{j}$ is the number of type $j$ cells).

${Q}_{ref}$ collapses the expression levels of a gene over multiple cells of the same cell type into one mean value. A more precise quantification of single-cell gene expression values is to infer their distributions. To simplify the model of single-cell gene expressions, we make two explicit assumptions. First, marker genes in the single-cell data are subdivided into groups. Second, the normalized expressions of marker genes in the same group are drawn from the same distribution. Both assumptions are valid as marker genes are selected according to the criteria that they are expressed in specific sample types or cell types. These assumptions enable us to estimate the expression value distributions of a small number of gene groups rather than all the marker genes separately. The reference signature distribution ${GP}_{ref}$ is an ${n}_{g}\times k\times I$ tensor for ${n}_{g}$ gene groups, $k$ cell types and $I$ intervals of gene expression values. ${GP}_{ref}(i,j,l)$ specifies the probability that the expression values of gene group $i$ in cell type $j$ fall in the ${l}^{th}$ interval. It is inadequate to directly estimate ${GP}_{ref}$ from $X$ because marker genes in the same group may have quite different scales of expression levels. To make the expression levels of all marker genes comparable, we rank-transformed the expression values of each gene and normalized the ranks into cumulative distribution function (cdf) values. In the normalized sc-RNASeq data matrix ${X}_{cdf}$, all entries take values in $[\text{0,1}]$ and the orders of entry values in each row are preserved from $X$. ${GP}_{ref}(i,j,:)$ specifies the probability mass function of normalized expression values of gene group $i$ and cell type $j$ in the interval $[\text{0,1}]$. We subdivided $[\text{0,1}]$ into $I$ intervals, identified genes ${\Lambda }_{i}$ belonging to group $i$ and cells ${S}_{j}$ belonging to type $j$, and collected the corresponding ${X}_{cdf}$ entries ${X}_{cdf}({\Lambda }_{i}, {S}_{j})$. ${GP}_{ref}(i,j,:)$ was obtained from ${X}_{cdf}({\Lambda }_{i}, {S}_{j})$ by kernel density estimation. To avoid minus infinity values of log-likelihood scores in the subsequent steps, all entries in ${GP}_{ref}$ need to be positive. We replaced zero entries in ${GP}_{ref}(i,j,:)$ with a small value $\epsilon$ and renormalized ${GP}_{ref}(i,j,:)$ to make them sum to 1 ($\epsilon =0.001$ in our analysis).

### Constructing reference signature matrices and distributions from the bulk-level data

When the sc-RNASeq data are absent, unannotated or of poor quality (such as the breast cancer and LGG data used in the present study), we have to construct the reference signature matrices and distributions from the bulk-level data alone. Complete deconvolution algorithms infer the signature matrix $Q$ and the mixture coefficients $P$ from the bulk-level data $E$ (Eq. 1). The reference signature matrix ${Q}_{ref}$ is the inferred signature matrix $Q$, yet the reference signature distributions ${GP}_{ref}$ cannot be obtained from the deconvolution outcomes. For incomplete deconvolution methods, both ${Q}_{ref}$ and ${GP}_{ref}$ need to be constructed from the bulk-level data before launching deconvolution. To construct ${Q}_{ref}$ and ${GP}_{ref}$ for incomplete methods we have to impose stronger hypotheses about the relations between the expression patterns of bulk-level sample subtypes and cell types. Below we describe the procedure of constructing the reference signature matrix ${Q}_{ref}$ and distribution ${GP}_{ref}$ of three simple models from the bulk-level RNASeq data of breast cancer or low-grade glioma.

Any model specifying the relations between sample subtypes and cell types has to calculate each entry in ${Q}_{ref}$ and ${GP}_{ref}$ from a subset of samples in the bulk-level data. To establish the bases of all possible models we derived three quantities from the bulk-level data: (1) partition of the normalized bulk-level data into a grid of gene groups and sample subtypes, (2) the mean expression value of each grid component, (3) quantization of the grid component mean expression matrix. Denote an $n\times m$ matrix $E$ the bulk-level RNASeq data of $n$ genes and $m$ samples. The first step is to normalize $E$ into a matrix ${E}_{cdf}$ by evaluating the cdf values of each row of $E$. This step is identical to the construction of ${X}_{cdf}$ from $X$. The second step is to partition ${E}_{cdf}$ into a grid of ${n}_{g}$ gene groups (row partition) and ${n}_{s}$ sample subtypes (column partition). Denote a $1\times n$ vector $\Gamma$ the gene group labels and a $1\times m$ vector $\Sigma$ the sample subtype labels, and ${\Lambda }_{i}\equiv \{g:{\Gamma }_{g}=i\}$ and ${\text{S}}_{j}\equiv \{s:{\Sigma }_{s}=j\}$ the gene group $i$ members and sample subtype $j$ members respectively. Each component ${E}_{cdf}({\Lambda }_{i},{\text{S}}_{j})$ of the grid contains the entries of ${E}_{cdf}$ belonging to gene group $i$ and sample subtype $j$. The breast cancer bulk-level data comprises three gene groups and four sample subtypes (basal, Her2-enriched, luminal A and luminal B), and the LGG data comprises three gene groups and three sample subtypes (Idh1 mutation with chromosome 1p/19q co-deletion, Idh1 mutation without the co-deletion, and wild type). The third step is to construct a grid-level expression data by taking the average of ${E}_{cdf}$ entries in each grid component: denote $G$ an ${n}_{g}\times {n}_{s}$ matrix and ${G}_{ij}\equiv \frac{1}{{|\Lambda }_{i}||{S}_{j}|}{\sum }_{a\in {\Lambda }_{i},b\in {S}_{j}}{E}_{cdf}(a,b)$. The fourth step is to quantize $G$ to an ${n}_{g}\times {n}_{s}$ matrix $C$ of trinary values indicating whether each grid component is up/down regulated or neither. For each gene group $1\le i\le {n}_{g}$, we extracted the ${E}_{cdf}$ entries belonging to gene group $i$: ${E}_{cdf}\left({\Lambda }_{i},:\right)\equiv \left\{{E}_{cdf}\left(a,b\right):a\in {\Lambda }_{i}, 1\le b\le m\right\}$, and calculated their mean ${\mu }_{{g}_{i}}$ and standard deviation ${\sigma }_{{g}_{i}}$. ${C}_{ij}=+1$ if ${G}_{ij}\ge {\mu }_{{g}_{i}}+{\sigma }_{{g}_{i}}$, ${C}_{ij}=-1$ if ${G}_{ij}\le {\mu }_{{g}_{i}}-{\sigma }_{{g}_{i}}$, and ${C}_{ij}=0$ otherwise. This quantization specifies whether the mean value of each grid component significantly deviates from the global mean value of the same gene group in positive or negative directions.

We then constructed the three models about the gene expression patterns underlying the unobserved cell types. These models are illustrative examples of simple hypotheses about the relations between sample subtypes and cell types but by no means an exhaustive list of possible gene expression patterns of cell types. ${M}_{1}$ stipulates that tumors of each subtype is dominated by one unique cell type, hence the gene expression profiles of cell types resemble the bulk-level data. For breast cancer data, ${M}_{1}$ assumes that the bulk samples of each subtype (basal-like, Her2-enriched, luminal A, luminal B) are dominated by one cell type. Hence the reference signature matrix and distribution of a cell type are estimated from the bulk-level data of the corresponding cancer subtype. The bulk-level expression data (METABRIC) are treated as the reference sc-RNASeq data to construct ${Q}_{ref}$ and ${GP}_{ref}$ (Fig. 4A, left panel). Entries in the signature matrix ${Q}_{ref}$ are the average of the corresponding entries of (gene, sample subtype) combinations in $E$: ${Q}_{ref}\left(i,j\right)=\frac{1}{| {\text{S}}_{j}|}{\sum }_{b\in {\text{S}}_{j}}E(i,b)$. Entries in ${GP}_{ref}$ are estimated by the corresponding entries of (gene group, sample subtype) combinations in ${E}_{cdf}$. For gene group $i$ and sample subtype $j$, we extracted the ${E}_{cdf}$ entries ${E}_{cdf}({\Lambda }_{i},{S}_{j})\equiv \left\{{E}_{cdf}\left(a,b\right):a\in {\Lambda }_{i}, b\in {S}_{j}\right\}$. We then applied kernel density estimation to ${E}_{cdf}({\Lambda }_{i},{S}_{j})$ and assessed the probability mass function ${p}_{M}(x)$ on intervals $0:\frac{1}{I}:1$. ${p}_{M}$ is a $1\times I$ vector and ${p}_{M}(x)\equiv \text{Pr}(\frac{x-1}{I}\le y\le \frac{x}{I})$ for $y\in {E}_{cdf}({\Lambda }_{i},{S}_{j})$. To avoid zero probability values in ${GP}_{ref}$, we replaced zero values in ${p}_{M}$ with a small but nonzero value $\epsilon$ ($\epsilon =0.001$ in our analysis) and renormalized ${p}_{M}$ to make the components sum to 1. Finally, we substituted the vector ${p}_{M}$ in ${GP}_{ref}(i,j,:)$. This estimation treats the bulk-level data as the single-cell data and equates sample subtypes and cell types. Hence the assumption of ${M}_{1}$ holds.

${M}_{2}$ stipulates that each cell type has high expressions in one gene group and low expressions in other gene groups. Therefore, we identified the bulk-level grid components corresponding to up or down regulation and assigned them to proper positions to assess ${Q}_{ref}$ and ${GP}_{ref}$. For breast cancer, ${M}_{2}$ assumes that there are three cell types. Each cell type has high expression values of one marker gene group and low expressions of the other two marker gene groups, and the three marker gene groups are enriched with cell cycle control, immune response, and estrogen response respectively. We then quantized the grid of bulk-level expression data of (gene groups, sample subtypes) into trinary values. To estimate the reference signature matrix and distribution of a marker gene in a gene group (for instance, cell cycle control) in the corresponding cell type (the cell type with high expressions of cell cycle control genes), we solicited the sample subtypes with high expressions of the marker gene group (basal-like, Her2-enriched, and luminal B) and estimated ${Q}_{ref}$ and ${GP}_{ref}$ from the selected bulk samples (Fig. 4A, left panel). Similarly, ${Q}_{ref}$ and ${GP}_{ref}$ of a cell cycle gene in a cell type with low expression of the cell cycle gene group are estimated from the bulk samples with low expressions of the cell cycle gene group (luminal A samples). For a gene group $i$, we identified two subsets of sample subtypes where the quantized grids $C$ had + 1 and − 1 values: ${\text{H}}_{i}^{+}\equiv \{j:C\left(i,j\right)=1\}$, ${\text{H}}_{i}^{-}\equiv \{j:C\left(i,j\right)=-1\}$. We then identified the samples whose subtypes belonged to ${\text{H}}_{i}^{+}$ and ${\text{H}}_{i}^{-}$ respectively: ${S}_{i}^{+}\equiv \left\{s:{\Sigma }_{s}\in {\text{H}}_{i}^{+}\right\}, {S}_{i}^{-}\equiv \left\{s:{\Sigma }_{s}\in {\text{H}}_{i}^{-}\right\}.$ For each gene $g\in {\Lambda }_{i}$ and sample subtype $j$, ${Q}_{ref}\left(g,j\right)=\frac{1}{|{S}_{j}^{+}|}{\sum }_{\{s\in {S}_{j}^{+}\}}E(g,s)$ if $j=i$, and ${Q}_{ref}\left(g,j\right)=\frac{1}{|{S}_{j}^{-}|}{\sum }_{\{s\in {S}_{j}^{-}\}}E(g,s)$ if $j\ne i$. In other words, ${Q}_{ref}\left(g,j\right)$ is the average of gene $g$ expressions over the up-regulated entries for cell type $i$ and the average of gene $g$ expressions over the down-regulated entries for other cell types. Similarly, the distribution ${GP}_{ref}(i,j=i,:)$ was estimated from the entries of gene group $i$ (${\Lambda }_{i}$) and the up-regulated samples ${S}_{i}^{+}$: ${E}_{cdf}\left({\Lambda }_{i},{S}_{i}^{+}\right):\{{E}_{cdf}\left(a,b\right):a\in {\Lambda }_{i}, b\in {S}_{i}^{+}\}$, and ${GP}_{ref}(i,j\ne i,:)$ was estimated from ${\Lambda }_{i}$ and the down-regulated samples ${S}_{i}^{-}$: ${E}_{cdf}\left({\Lambda }_{i},{S}_{i}^{-}\right):\{{E}_{cdf}\left(a,b\right):a\in {\Lambda }_{i}, b\in {S}_{i}^{-}\}$. Therefore, ${GP}_{ref}(i,j=i,:)$ assigns high probability mass to high expression values and ${GP}_{ref}(i,j\ne i,:)$ assigns high probability mass to low expression values, which meets the assumption of ${M}_{2}$.

${M}_{3}$ serves as a negative control of ${M}_{1}$ as the two models have the same number of cell types and ${M}_{3}$ is obtained from ${M}_{1}$ by rearranging rows and columns to maximize the difference. In breast cancer data, we permuted entries in each row of $G$ independently and exhausted all ${24}^{3}=13824$ permutations. Each permutation $\psi$ induced a matrix ${G}_{\psi }$. We then exhausted all $24$ column permutations of ${G}_{\psi }$ and  found the best alignment with $G$. The resulting grid matrix $\widehat{G}$ yields the max–min ${L}_{2}$-norm difference from $G$:

5 $$\hat{G} = \arg \mathop {\max }\limits_{\psi } \mathop {\min }\limits_{\phi } |G - G_{\phi \circ \psi } |_{2} .$$

$\psi$ denotes a combination of independent permutations of entries in each row, $\phi$ denotes a column permutation, and $\phi \circ \psi$ denotes a composition of independent row entry permutations followed by a column permutation. The optimal permutation $\widehat{\phi }\circ \widehat{\psi }$ assigns a grid component in $G$ to another grid component in $\widehat{G}$. Therefore, we redefined ${S}_{j}$ for sample subtype $j$ and ${S}_{i}^{+}$ and ${S}_{i}^{-}$ for gene group $i$ according to $\widehat{\phi }\circ \widehat{\psi }$ and re-calculated ${Q}_{ref}$ and ${GP}_{ref}$ following the procedure for constructing ${M}_{1}$.

In LGG data, we found that $\widehat{G}$ yielded one column of low expressions for all gene groups. This column (cell type) can fit many cells with sparse nonzero entries, hence will distort the log-likelihood scores and make ${M}_{3}$ more favorable. To circumvent this distortion, we manually re-assigned grids in $G$ to form $\widehat{G}$ such that each cell type consisted of at least one up-regulated gene group and one down-regulated gene group. The ${M}_{3}$ signature matrix of the LGG data is displayed in Supplementary file 3: Figure S3A.

### Deconvolving bulk-level gene expression data

A (forward) deconvolution algorithm factorizes a bulk-level gene expression matrix $E$ into the product of the cell type signature matrix $Q$ and the sample mixture coefficient matrix $P$ (Eq. 1). Incomplete algorithms require $Q$ as explicitly given or derived from an external source. We used ${Q}_{ref}$ generated from Sect. "Generating reference signature matrices and distributions from the single-cell data" or "Constructing reference signature matrices and distributions from the bulk-level data". Complete algorithms return both $Q$ and $P$. Here we selected nine deconvolution algorithms: DeconRNASeq [12], lsfit [31], DWLS [14], NMF [3], two versions of deconf (original and fast) [32, 33], bMIND [34], RADs [35], and Scaden [36]. Scaden was a supervised deep learning algorithm that required labels of sc-RNASeq data, hence was not applicable for our cancer datasets due to lack of cancer cell type annotations. The first and last three are incomplete methods and the remaining three are complete methods. An R package CellMix [33] includes deconf and lsfit implementations, and the remaining algorithms have their own R or Python packages. The complete methods differ in their respective cost functions (e.g. Euclidean distance between the target matrix and the NMF estimate in deconf original and Kullback–Leibler divergence in Brunet’s NMF), their algorithms (multiplicative or least squares based), stopping criteria, and the ways the non-negativity and scaling constraints are enforced onto the signature and the mixture coefficient matrices. Thus, two algorithms (Brunet’s NMF and deconf) represent two significantly different methods while the two versions of deconf represent the class of similar methods. All the incomplete methods are variations of non-negative least squares optimization algorithm and have different approaches to estimating cell proportions from signatures. This combination of both similar and dissimilar methods can offer additional insights into the performance of the backward deconvolution framework.

### Constructing probabilistic graphical models of single-cell gene expressions

As mentioned in Sect. "Fitting single-cell gene expression data with probabilistic graphical models constructed from forward deconvolution outcomes", the sc-RNASeq data generation process is represented as a probabilistic graphical model with two families of conditional probabilities $P\left(\pi |s\right)$ and $P(x|$ $\gamma ,\pi )$. We propose a procedure to construct $P\left(\pi |s\right)$ and $P(x|$ $\gamma ,\pi )$ from the forward deconvolution outcome based on the following assumptions: (1) expressions from the same gene group and cell type are drawn from the same underlying distribution, (2) samples of the same subtype possess similar cell type compositions, (3) each reference or inferred signature vector (a column in $Q$) can be viewed as the expression profile of a virtual bulk sample; after rescaling the reference signature value of each gene is in the expression value range of the same gene in the bulk-level data, (4) the expression patterns in the bulk-level and single-cell RNASeq data are preserved after conversion into cdf values. Assumptions 1 and 2 simplify the models by collapsing the parameters pertaining to members of a gene group and a sample subtype. They are sensible if adequate marker genes of each cell type or subtype are selected from data. Assumption 3 ensures we can reasonably infer $P(x|$ $\gamma ,\pi )$ by comparing rescaled $Q$ values with bulk-level data values. It is sensible since bulk-level samples typically have small variations in the ${L}_{2}$-norms of their expression profiles. Assumption 4 ensures we can employ the models inferred from the bulk-level data to calculate the log-likelihood scores of single-cell data even though the two datasets may have very different scales. It is valid since the expression patterns are largely preserved after rank transformation [37].

$P\left(\pi |s\right)$ is directly estimated from the mixture coefficient matrix $P$. Denote ${S}_{i}$ the bulk samples belonging to subtype $i$, then $P\left(\pi =j|s=i\right)=\frac{{\sum }_{l\in {S}_{i}}{P}_{jl}}{{\sum }_{{j}{\prime}=1-k,l\in {S}_{i}}{P}_{j{\prime}l}}$.

Complete algorithms report signature matrices $Q$, and incomplete algorithms take the reference signature matrices as inputs (${Q=Q}_{ref}$).

The signature matrix $Q$ reports the average expression value of each gene in each cell type. $P(x|$ $\gamma ,\pi )$ reports the distribution of expression values conditioned on a gene group and cell type. Direct estimation of $P(x|$ $\gamma ,\pi )$ from $Q$ is not stable due to the small number of entries to assess cdf values. Each gene in $Q$ has only a small number (the number of cell types) of expression values. Hence the rank transform of rows in $Q$ gives a very crude quantization of signature matrix values. To mitigate this problem, we calculated the cdf values of $Q$ entries in terms of a much larger pool $E$ (the bulk-level RNASeq data) rather than $Q$ itself. In other words, the cdf value of an entry ${Q}_{ij}$ was calculated by comparing ${Q}_{ij}$ with the entries in the ${i}^{\text{th}}$ row of $E$, rather than the entries in the ${i}^{\text{th}}$ row of $Q$. The rank-transformed matrix of $Q$ was used to estimate $P(x|$ $\gamma ,\pi )$. However, entries in $Q$ and $E$ are not necessarily comparable since $Q$ may be derived from the single-cell RNASeq data which often has a very different scale than $E$. We adopted assumption (3) in Sect. "Constructing probabilistic graphical models of single-cell gene expressions" to rescale each $Q$ column by the median column norm of $E$, and rank-transformed the rescaled $Q$ into cdf values. The following procedure was executed.

1. Rescaled each column of $Q$ separately to make the signature matrix entries have comparable values as the bulk expression data $E$. Calculated the ${L}_{2}$-norm of each column in $E$ and denoted them $Z=\{{z}_{1},\cdots ,{z}_{m}\}$, and the ${L}_{2}$-norm of each column in $Q$ and denoted them $Y=\{{y}_{1},\cdots ,{y}_{k}\}$. Denoted the median of $Z$ as $\overline{z }$. Rescaled column $j$ of $Q$ by multiplying it by a factor ${r}_{j}=\frac{\overline{z}}{{y }_{j}}$: ${\widehat{Q}}_{*,j}={r}_{j}\cdot {Q}_{*,j}$. Each column of the rescaled signature matrix $\widehat{Q}$ had an identical ${L}_{2}$-norm $\overline{z }$ as median ${L}_{2}$-norm over all bulk-level samples, implying that the cell type gene expression profiles were comparable to the bulk sample gene expression profiles after rescaling.
2. Normalized the rescaled signature matrix into cdf values. For each entry ${\widehat{Q}}_{ij}$ in $\widehat{Q}$ (gene $i$ and cell type $j$), found row $i$ in $E$ (expressions of gene $i$ in all bulk samples) and denoted it as ${E}_{i,*}$. Calculated the cdf value of ${\widehat{Q}}_{ij}$ in ${E}_{i,*}$ as the fraction of ${E}_{i,*}$ entries with values $\le {\widehat{Q}}_{ij}$, and denoted its value as ${W}_{ij}$. Repeated the same procedure for all genes and prototypes and completed the signature cdf value matrix $W$. $W$ entry values range in $[\text{0,1}]$ and have dimension $n\times k$.
3. Estimated $P(x|$ $\gamma ,\pi )$ from $W$. Each column in $W$ denoted the normalized expression profile of a cell type. For each $\gamma =i$ and $\pi =j$, solicited the $W$ entries for gene group $i$ and cell type $j$. $P(x|$ $\gamma ,\pi )$ was the density estimate of the selected entries. Similar to prior procedures, we subdivided $[\text{0,1}]$ into $I$ equal intervals $0:\frac{1}{I}:1$ and applied kernel density estimation to calculate the probability value of each interval. We also replaced zeros in $P(x|$ $\gamma ,\pi )$ with a small value $\epsilon$ and re-normalized the entries to make them legitimate conditional probabilities.

The input ${Q}_{ref}$ of each incomplete algorithm is often accompanied with a reference signature distribution ${GP}_{ref}$. We also use ${GP}_{ref}(\gamma ,\pi ,:)$ as a more precise model of $P(x|$ $\gamma ,\pi )$. Consequently, each incomplete algorithm reports two types of $P(x|$ $\gamma ,\pi )$: ${P}_{Q}(x|$ $\gamma ,\pi )$ derived from $Q$ and ${P}_{{GP}_{ref}}(x|$ $\gamma ,\pi )$ derived from ${GP}_{ref}$, while each complete algorithm reports only ${P}_{Q}(x|$ $\gamma ,\pi )$.

### Comparing the deconvolution algorithms in fitting sc-RNASeq data

Once the parameters of $P\left(\pi |s\right)$ and $P(x|$ $\gamma ,\pi )$ were inferred from the deconvolution outcomes of bulk-level RNASeq data, we substituted them into Eqs. 2–4 to evaluate the joint likelihood, marginal likelihood, and BIC score of the sc-RNASeq data respectively. For each incomplete method we had two sets of estimated $P(x|$ $\gamma ,\pi )$ tables (${P}_{Q}(x|$ $\gamma ,\pi )$ and ${P}_{{GP}_{ref}}(x|$ $\gamma ,\pi )$) to evaluate the likelihood and BIC scores.

The degree of freedom $D$ in the BIC score (Eq. 4) is determined by the number of independent entries in $P\left(\pi |s\right)$ and $P(x|$ $\gamma ,\pi )$. For instance, in breast cancer data there are 4 sample subtypes ($s$ values), 3 gene groups ($\gamma$ values), and 10 intervals of gene expression values ($x$ values). The two models described in Sect. "Constructing reference signature matrices and distributions from the bulk-level data" have 4 and 3 cell types ($\pi$ values) respectively. Therefore, model 1 has $4\times 3+3\times 4\times 9=120$ free parameters, and model 2 has $4\times 2+3\times 3\times 9=89$ free parameters. Complexity penalty is not needed when all models in a dataset have the same degree of freedom.

Single-cell RNASeq data is filled with many zero entries. Abundant zero entries implicate their dominant contribution to the BIC scores. We propose a simple criterion for inclusion or exclusion of zero entries in log-likelihood score computation according to the concentration or depletion of zero entries in the grids of (gene group, sample subtype) combinations. The procedure and decision for zero entry inclusion/exclusion of the datasets are reported in Sect. "Handling zero entries in calculating log-likelihood scores of the sc-RNASeq data".

For incomplete methods in annotated sc-RNASeq data, backward deconvolution uses the single-cell data to both infer the models and evaluate the log-likelihood scores. To avoid double usage of the single-cell data, we split sc-RNASeq data into the sets for constructing the reference signature matrix and distribution (training data) and evaluating the log-likelihood score of the model (test data). In our experiments we assigned the same number of cells to the training and test data.

We selected the deconvolution model that yielded the highest BIC score on the single-cell data and reported $Q, P, P\left(\pi |s\right), P(x|\gamma ,\pi )$, and the cell type assignments in the single-cell data. To infer the type of a cell $j$, we calculated the posterior likelihood score conditioned on each possible cell type:

6 $$L\left( {\pi_{j} = u|s\left( j \right) = \sigma ,X} \right) \propto P(\pi = u|s = \sigma )\mathop \prod \limits_{l} P(x_{jl} |\gamma = \gamma_{l} ,\pi = u).$$

where $s\left(j\right)$ denotes the sample subtype of cell $j$, index $l$ is over all genes, and ${x}_{jl}$ denotes the normalized expression of gene $l$ in cell $j$. Cell $j$ is assigned to the cell type of the highest posterior likelihood.

### Handling zero entries in calculating log-likelihood scores of the sc-RNASeq data

Correctly handling the missing values in the sc-RNASeq data is crucial to ensure the reliability of downstream analyses. There are two general approaches handling dropout (zero) entries in the sc-RNASeq data. The first approach imputes the dropout entries. Numerous imputation techniques based on clustering, deep learning algorithms, or fitting various statistical models underlying the observed expression values have been proposed [38–40]. The imputation approach infers zero entries with information of nonzero entries hence suffers from two shortcomings: the imputed values are based on specific assumptions of the data, and the information of zero entries is discarded. The second approach treats missing values as informative biological signals, hence uses them for inferring relevant information for downstream analyses. It was shown that the distribution of dropout entries can be used for cell type identification and trajectory inference [41, 42], feature selection tasks [43], data projections [44], and others. In line with the second approach, we proposed a simple criterion for including or excluding zero entries in computing the log-likelihood scores of a sc-RNASeq dataset. Intuitively, if zero entries are strongly enriched or depleted in specific (gene group, cell type) combinations, then they likely reflect low or high values in the gene expression signatures hence should be included in log-likelihood calculation. Conversely, if zero entries are not strongly enriched in specific (gene group, cell type) combinations, then they likely reflect random noise due to dropouts hence should be discarded. This intuitive criterion is translated into the following procedure. First, the sc-RNASeq data was subdivided into a grid of gene groups and cell types. If cell types were not annotated, then cells were subdivided by the subtypes of their bulk samples. Second, in each grid component we counted the number of zero entries. Third, we randomly permuted cells in the data 10,000 times and counted the number of zero entries in each grid component and each random permutation. Fourth, in each grid we calculated the mean and standard deviation of zero entry counts over 10,000 random permutations. Fifth, we counted the grid components whose zero entry counts were above or below the confidence interval of six standard deviations from the mean in the permuted data. Sixth, if the number of grid components with enriched or depleted zero entries constituted a substantial fraction of the total number of grid components (0.25 in our study), then we included zero entries in computing log-likelihood scores. Otherwise we excluded zero entries in computing log-likelihood scores.

Supplementary file 4: Table S1 reports the numbers of grids with enriched or depleted zero entries in each sc-RNASeq dataset. All but the two breast cancer datasets contain more than 25% of grids with enriched or depleted zero entries. Hence we excluded zero entries in log-likelihood score computation for the two breast cancer datasets and included zero entries for all other datasets.

### Selecting marker genes and cells from four single-cell and bulk-level datasets

We validated the backward deconvolution framework in five transcriptomic datasets: (1) the mouse sc-RNASeq data of 5 cell types and artificial mixtures of these data as the virtual bulk-level data, (2) the mouse bulk-level and single-cell RNASeq data of 4 tissue types and the 9 constituting cell types, (3) one bulk-level and one single-cell RNASeq datasets of human brain regions from Autism Spectrum Disorder (ASD) subjects and normal controls, (4) one bulk-level and two single-cell RNASeq datasets of breast cancer, (5) one bulk-level and one single-cell RNASeq datasets of low-grade gliomas (LGG). Below we describe the procedure of selecting marker genes of each dataset.

#### In-silico mixture of mouse sc-RNASeq data

We generated bulk level in-silico mixtures from single-cell RNASeq data of a mouse gene expression database [45]. We selected cells from five different cell types—oligodendrocytes, T cells, lung endothelial cells, hepatocytes, and fibroblast cells—along with the genes that were differentially expressed in those cell types, which we denoted as marker genes. These cell types were chosen primarily because their numbers of cells were relatively abundant and their expression patterns were relatively distinct. A marker gene of a cell type meets two criteria: (1) it has nonzero expressions in at least 75% of the cells of the target cell type and in at least 60 cells of each of the remaining cell types, and (2) it has a p-value < 0.05 for the one-tailed unpaired t-test between the target cell type and each of the remaining cell types. In total, we selected 226, 163, 92, 84, and 78 marker genes (for a total of 643) and 713, 375, 324, 196, and 1,082 cells (for a total of 2,690) for each cell type, respectively.

#### Mouse bulk-level and single-cell RNASeq data

The bulk-level and single-cell RNASeq data from the Tabula Muris Senis database were used [46]. We selected four tissue types: fat, heart, limb, and liver, and solicited cells from the same tissue types in the mouse single-cell RNASeq data. The four tissue types constituted nine cell types: endothelial cells, fibroblast/mesenchymal cells, epithelial cells, immune cells, smooth muscle cells, skeletal cells, endocardial cells, Schwann cells and hepatocyte cells. The diverse family of immune cells (e.g., T cells, B cells, macrophages, etc.) was collapsed into one cell type in order to reduce the number of cell types and hence simplify deconvolution. Fibroblast and mesenchymal cells were also collapsed into one cell type as they had very similar expression profiles.

For each gene, we extracted the cells of each type and calculated the mean and standard deviation of their sc-RNASeq data in the nine cell types. The signal-to-noise ratio between cell types $i$ and $j$ was the difference of their mean values ${m}_{i}$ and ${m}_{j}$ normalized by their standard deviations ${\sigma }_{i}$ and ${\sigma }_{j}$: ${SNR}_{ij}=\frac{2({m}_{i}-{m}_{j})}{({\sigma }_{i}+{\sigma }_{j})}.$ A gene was selected as a marker gene of cell type $i$ if its ${SNR}_{ij}\ge 0.5$ for all $j\ne i$. There were totally 4439 marker genes and 33,777 cells in the single-cell RNASeq.

#### ASD bulk-level and single-cell RNASeq data

The bulk-level and single-cell RNASeq data of brain regions from ASD patients and normal controls were from distinct sources. The sc-RNASeq data [47] comprises 104,559 cells from ASD and normal subjects of two brain regions: anterior cingulate cortex (ACC) and prefrontal cortex (PFC). Together these cells belong to 15 simplified cell types: IN-PV, IN-SST, IN-SV2C, IN-VIP, L2/3, L4, L5/6, L5/6-CC, Neu-NRGN, Neu-mat, Microglia, Astrocytes (AST), Endothelial, Oligodendrocyte Progenitor Cells (OPC), and mature Oligodendrocytes. Two types of astrocytes were combined into one astrocyte cell type, and two types of Neu-NRGN cells were combined into one Neu-NRGN cell type. The first 10 cell types are neuron cells and the remaining 5 are supporting cells. The bulk-level RNASeq data [48] comprises 104 samples from ASD and normal subjects of three brain regions: Brodmann areas 10 (decision making), 19 (vision processing) and 44 (motor aspect of speech).

We identified the marker genes with differential expressions in each of the 15 cell types in the sc-RNASeq data. Velmeshev et al. [47] reported the differentially expressed genes between diagnostic phenotypes (ASD vs. normal), brain regions and individuals. We collected these genes as the candidates for the marker genes. For each candidate gene, we calculated the mean expression values over the cells of each type and sorted the cell types accordingly. The overwhelming score of a gene was the mean expression value of the top cell type minus the sum of mean expression values of all other cell types. We selected the genes whose overwhelming scores $\ge 0$. If a cell type had less than 20 marker genes according to this condition, we then relaxed the criterion and selected the top-ranking genes according to their overwhelming scores. 500 marker genes were selected accordingly.

The brain regions covered in the single-cell and bulk-level data are not identical. To make likelihood evaluation feasible we have to establish a mapping between bulk-level sample subtypes and single-cell sample subtypes. There are four sample subtypes in the sc-RNASeq data (two phenotypes multiply two brain regions) and six sample subtypes in the bulk-level data (two phenotypes multiply three brain regions). We exhausted all 6 possible assignments from three brain areas in the bulk-level data to two brain areas in the single-cell data. For each possible assignment, we evaluated the ${\mathcal{L}}_{2}$ scores of 6 incomplete methods, and identified the assignment which gave the highest ${\mathcal{L}}_{2}$ scores in most of the methods. The assignment ACC $\to$ BA19, PFC $\to$ BA44 was chosen accordingly.

#### Breast *cancer* bulk-level and single-cell RNASeq data

We extended the PAM50 genes [49] with a procedure described in Tiong et al*.* 2022 [50]. The PAM50 genes were divided into three groups by k-means clustering on the METABRIC gene expression data. The three groups were enriched in cellular functions related to cell cycle control, immune responses, and estrogen receptors respectively. To extend the PAM50 gene list, we calculated correlation coefficients of other genes in TCGA-BRCA and METABRIC data with the PAM50 genes, and averaged the correlation coefficients over the members of each PAM50 group. Candidate genes were sorted by the maximum of group-level average correlation coefficients in each dataset (TCGA-BRCA and METABRIC) separately. A total of 200 genes were selected (intersection of genes sorted by the maximum group-level average correlation coefficients from both datasets), and further filtered down to 127 genes after selecting for genes having > 70% valid entry in the single cell data.

The breast cancer sc-RNASeq datasets contain non-cancer cells and cancer cells with sparse valid (nonzero) entries. We cleaned the data by considering only cancer cells with $\ge \frac{1}{3}$ of the valid entries among the marker genes since the proportions of normal cells in single-cell RNASeq data were unlikely those from tumors. There were 281 and 12,019 selected cancer cells in the two single-cell RNASeq datasets respectively. In addition, Supplementary file 4: Table S1 indicates that the breast cancer sc-RNASeq datasets have more disperse distributions of zero entries. Therefore, zero entries were discarded when evaluating the log-likelihood scores. Penalty terms of model complexity in Eq. 4 were added since the models had different numbers of parameters.

To provide more direct evidence supporting superiority of $${M}_{1}$$ to fit the breast cancer sc-RNASeq data, we compared the clustering outcomes of an independent breast cancer sc-RNASeq dataset with those of two virtual sc-RNASeq datasets simulated from $${M}_{1}$$ and $${M}_{2}$$. We downloaded the GSE161529 data comprising 305,157 tumor cells from 45 patients [51]. Due to hardware limitations, a total of 50,000 cells (cells with the most non-zero entries) from 33 patients were initially selected for our analysis. Seurat preprocessing pipeline [52] further filtered out the cells to the final number of 14,004 cells, which were subsequently used for clustering and t-SNE visualization. The virtual sc-RNASeq data of 40 patients (10 patients for each subtype, 1000 cells for each patient) were generated by sampling from the probabilistic graphical models (Eq. 2). $$P(x|$$ $$\gamma ,\pi )$$’s were inferred from the $${M}_{1}$$ or $${M}_{2}$$ signature matrix, and $$P(\pi |$$ $$s)$$’s were estimated from the METABRIC bulk-level data by DWLS. The simulated cells were also clustered using Seurat.

#### Low-grade glioma bulk-level and single-cell RNASeq data

Marker genes for glioma were derived based on our previous observation [50, 53] that 3 LGG subtypes: IDH-mutant with chromosome 1p19q co-deletion (CoDel), IDH-mutant with no chromosome 1p19q co-deletion (NoCoDel), and IDH wildtype (WT) possess highly expressed genes in three gene groups. Corresponding MSigDB gene sets of the enriched cellular functions were tested for subtype-specific differential expression (t-test) in TCGA LGG gene expression data, further narrowed down to genes having > 70% valid entry in the single cell data, resulting in a final list of 61 genes.

#### Introducing variations to artificial mixtures bulk-level data

One dataset in our analysis is in-silico mixture of mouse sc-RNASeq data: the virtual bulk-level data was computationally generated by sampling and mixing the experimental single-cell data. A straightforward process of generating the in-silico bulk-level data is to fix the mixture coefficients and exert no additional noise beyond the experimental single-cell data. However, since the mouse sc-RNASeq data was relatively clean, nearly all deconvolution algorithms we picked successfully decomposed the virtual data generated from this process. To test the capacity of the deconvolution algorithms, we introduced two types of variations from the aforementioned artificial mixtures: fluctuations of the mixture coefficients vector and additive noise to the virtual bulk expression data. Denote $\overrightarrow{\mu }\equiv ({\mu }_{1},\cdots ,{\mu }_{5})$ the ideal mixture coefficient vector of a sample subtype. Each vector has five components indicating the mixture coefficient of five cell types. We created variations of the mixture coefficient vectors from $\overrightarrow{\mu }$ by sampling the mixture coefficient vectors $\overrightarrow{\lambda }$ from a Dirichlet distribution:

7 $$P\left(\overrightarrow{\lambda }|\overrightarrow{\mu },\eta \right)\equiv \frac{1}{Z\left(\overrightarrow{\mu },\eta \right)}\prod_{i=1}^{5}{\lambda }_{i}^{\eta {\mu }_{i}-1}.$$

Parameters $\overrightarrow{\mu }$ and $\eta$ specify the mean mixture coefficient vector and scale of concentration around the mean, and $Z\left(\overrightarrow{\mu },\eta \right)$ is a normalization constant. A large $\eta$ makes $\overrightarrow{\lambda }$ narrowly concentrated around the mean, and a small $\eta$ makes $\overrightarrow{\lambda }$ disperse in a wide range.

After $\overrightarrow{\lambda }$ was sampled from Eq. 7 we sampled $(\left[{\lambda }_{1}N\right],\cdots ,\left[{\lambda }_{5}N\right])$ cells from the sc-RNASeq data with replacement and calculated the average expression profile of the marker genes over the sampled cells. Denote $\overrightarrow{x}$ this average expression profile vector. We then introduced nonnegative noise to the virtual bulk sample by augmenting $\overrightarrow{x}$ with a random value sampled from a gamma distribution. The noisy expression of gene $i$ in the virtual bulk sample is:

8 $${y}_{i}={x}_{i}+f\left(i,\overrightarrow{\mu }\right)\delta {\epsilon }_{i}, {\epsilon }_{i} \sim\Gamma \left(0.1{x}_{i},0.1\right).$$

${\epsilon }_{i}$ was sampled from a gamma distribution with shape parameter $\alpha =0.1{x}_{i}$, rate parameter $\beta =0.1$ and mean $\frac{\alpha }{\beta }={x}_{i}$, and the noise ${\epsilon }_{i}$ was diminished by a factor $\delta$. $f(i,\overrightarrow{\mu })$ is an indicator function of the condition that gene $i$ is a marker gene of the dominant cell type(s) of the ideal mixture coefficients vector $\overrightarrow{\mu }$. This noise is more challenging for deconvolution algorithms as it lifts the expression values of non-target cell types only and hence blurs the differential expressions between target and non-target cell types. The level of noise was controlled by the diminishing factor $\delta$. We considered two $\eta$ values 50, 3 and two $\delta$ values 0, 0.7, hence generated 4 sets of artificially mixed bulk data. The data of $\eta =50, \delta =0$ has no fluctuation of mixture coefficients vectors and no artificial noise of expression data, hence should be accurately deconvolved by most reasonable algorithms. The data of $\eta =3, \delta =0.7$ has the highest levels of mixture coefficient fluctuation and gene expression noise, hence will yield different results from different deconvolution algorithms.

## Results

We validated backward deconvolution using nine forward deconvolution algorithms (Sect. "Deconvolving bulk-level gene expression data") and four single-cell and bulk-level transcriptomic datasets (Sect. "Selecting marker genes and cells from four single-cell and bulk-level datasets"). In the two datasets of mouse tissues and the dataset of ASD brains, the cell types were annotated and the accuracy rates of cell type assignments were calculated. Consequently, we demonstrated validity of backward deconvolution by showing that the log-likelihood scores of nine deconvolution algorithms were correlated with several existing accuracy metrics. In the two datasets of human tumors, only normal cells were annotated with cell types (stromal cells, T cells, etc.), but cancer cells were not further annotated with refined cell types. Since our analysis focused on cancer cells, the accuracy rates of cell type assignments were inaccessible. Therefore, we adopted both indirect and direct approaches to validate backward deconvolution in cancer data. We proposed three simple hypotheses specifying the relation between tumor subtypes (which were annotated) and cancer cell types (which were not annotated), employed these hypotheses to construct the reference signature matrices and distributions, and compared their BIC scores of nine deconvolution algorithms. Intriguingly, one hypothesis was persistently superior across the deconvolution algorithms and datasets. Furthermore, we clustered and visualized an independent breast cancer sc-RNASeq dataset and compared the clustering results with those of two virtual datasets simulated from two models. The virtual data simulated from the superior model of the indirect approach also better resembled the real sc-RNASeq data compared to an alternative model. The results didn’t truly substantiate the favorable hypothesis but at least indicated it better fit the single-cell data in our analysis.

### In-silico mixture of mouse sc-RNASeq data

We downloaded the single-cell RNASeq data of the Tabula Muris database [45], selected 2690 cells from five cell types—oligodendrocytes, T cells, lung endothelial cells, hepatocytes, and fibroblast cells—and 643 marker genes which were differentially expressed in one cell type (see Sect. "Selecting marker genes and cells from four single-cell and bulk-level datasets" for the criteria of selecting marker genes and cells). We first constructed virtual bulk-level data by sampling and mixing the sc-RNASeq data in silico. Because both cell type annotations in the single-cell data and mixture coefficients of bulk-level data were available, we could directly relate accuracies in these two aspects with the log-likelihood scores derived from backward deconvolution.

We considered 16 mixture coefficient vectors (subtypes) of the 5 cell types (Supplementary file 1: Figure S1A, top-left panel). Five vectors had one dominant cell type (90%) and equal proportions for other cell types. Ten vectors had two dominant cell types (45% each). One vector had an equal proportion of each cell type. Each subtype constituted 100 bulk samples, and each sample was mixed from $N=1000$ randomly selected cells of the sc-RNASeq data. We introduced two types of variations to the artificial mixtures: fluctuations of the mixture coefficient vector (parametrized by scale of concentration $\eta$) and additive noise to the virtual bulk expression data (parametrized by noise diminishing factor $\delta$). The procedure of introducing variations to artificial mixtures is described in Sect. "Introducing variations to artificial mixtures bulk-level data". We considered two $\eta$ values 50, 3 and two $\delta$ values 0, 0.7, hence generated 4 sets of artificially mixed bulk data.

We calculated the log-likelihood scores for nine deconvolution algorithms. For each incomplete method, we calculated two log-likelihood scores of the models derived from $Q$ (${\mathcal{L}}_{1}$) and ${GP}_{ref}$ (${\mathcal{L}}_{2}$). For each complete method, we calculated ${\mathcal{L}}_{1}$ only.

Table 1 reports the log-likelihood scores for nine deconvolution algorithms on the four in-silico mixture datasets. We also report the sums of ${L}_{2}$-norms of the differences between the true and inferred mixture coefficient vectors, and those between the conditional probabilities $P(x|$ $\gamma ,\pi )$ constructed from the reference signature distribution (${P}_{{GP}_{ref}}(x|$ $\gamma ,\pi )$) and from the signature matrix (${P}_{Q}(x|$ $\gamma ,\pi )$). These two metrics reflect deviations from the ground truth in two aspects of decomposition (mixture coefficients and signature matrices/distributions).

**Table 1:** Inference results on four in-silico mixture datasets of mouse gene expressions

Dataset	Algorithm	${\mathcal{L}}_{1}$	${\mathcal{L}}_{2}$	Mixcoeffdiff	GPdiff	Corr (${\mathcal{L}}_{1}$, mixcoeffdiff)	Corr (${\mathcal{L}}_{2}$, mixcoeffdiff)	Corr (${\mathcal{L}}_{1}$, GPdiff)
50,0	DeconRNASeq	− 249,393,951	− 224,086,862	268.2334	4.7404	− 0.53410 (− 0.9843)	− 0.13078 (− 0.9763)	− 0.99937
	lsfit	− 248,591,799	− 223,776,456	37.8378	4.7404			
	DWLS	− 248,589,313	− 223,776,154	31.4589	4.7404			
	bMIND	− 248,752,114	− 223,697,645	434.8860	4.7404			
	RADs	− 248,573,045	− 223,758,072	72.7437	4.7404			
	Scaden	− 248,592,714	− 223,786,648	45.5412	4.7404			
	NMF	− 253,602,492		86.3161	4.8995			
	deconf_original	− 247,489,589		221.4328	4.3261			
	deconf_fast	− 252,928,470		326.5905	4.8154			
50,0.7	DeconRNASeq	− 241,681,325	− 223,623,107	97.3704	2.9296	0.62184 (0.9745)	− 0.33375 (− 0.9998)	− 0.79777
	lsfit	− 241,677,422	− 223,623,064	101.1241	2.9296			
	DWLS	− 241,646,421	− 223,621,886	77.6873	2.9296			
	bMIND	− 241,799,095	− 224,015,260	302.5663	2.9296			
	RADs	− 241,680,501	− 223,622,941	97.8673	2.9296			
	Scaden	− 241,509,933	− 223,718,001	805.0240	2.9296			
	NMF	− 240,774,724		989.7428	2.0442			
	deconf_original	− 243,807,979		855.6803	2.9779			
	deconf_fast	− 245,313,037		906.7191	2.6817			
3,0	DeconRNASeq	− 254,944,515	− 223,865,618	223.8699	4.1406	− 0.02364 (− 0.9403)	0.54718 (− 0.8879)	− 0.96092
	lsfit	− 254,870,022	− 223,822,966	35.2018	4.1406			
	DWLS	− 254,869,417	− 223,823,925	30.0576	4.1406			
	bMIND	− 254,842,203	− 223,749,266	410.4809	4.1406			
	RADs	− 254,856,595	− 223,808,415	67.3426	4.1406			
	Scaden	− 254,860,336	− 223,825,788	55.6792	4.1406			
	NMF	− 261,474,788		93.1494	4.2554			
	deconf_original	− 249,225,745		249.5048	4.1188			
	deconf_fast	− 263,147,812		263.7308	4.2307			
3,0.7	DeconRNASeq	− 240,404,254	− 223,702,592	95.0107	2.4743	0.99382 (0.9941)	− 0.99560 (− 0.9997)	− 0.99976
	lsfit	− 240,402,966	− 223,702,534	98.4294	2.4743			
	DWLS	− 240,392,342	− 223,702,650	81.3453	2.4743			
	bMIND	− 240,373,460	− 223,718,322	315.6408	2.4743			
	RADs	− 240,404,875	− 223,701,840	69.7891	2.4743			
	Scaden	− 240,287,880	− 223,791,143	941.3429	2.4743			
	NMF	− 250,257,532		1078.3727	2.9945			
	deconf_original	− 239,159,412		1012.1345	2.7048			
	deconf_fast	− 243,543,274		996.3748	2.8248			

Each dataset is specified by parameters $\left( {\eta ,\delta } \right)$. The models derived from nine algorithms are employed to evaluate log-likelihood scores. ${\mathcal{L}}_{1}$ and ${\mathcal{L}}_{2}$ denote the log-likelihood scores using $Q$ and $GP_{ref}$ to estimate $P(x|\gamma ,\pi )$. mixcoeffdiff denotes the sum of square errors between true and estimated mixture coefficients. GPdiff denotes the sum of square errors between true and estimated $P(x|\gamma ,\pi )$ tables derived from $GP_{ref}$ and $Q$ respectively. corr(${\mathcal{L}}_{1}$,mixcoeffdiff) and corr(${\mathcal{L}}_{2}$,mixcoeffdiff) denote the correlation coefficients between ${\mathcal{L}}_{1}$ (${\mathcal{L}}_{2}$) scores and mixcoeffdiff among the six incomplete methods. The values in the parentheses denote the correlation coefficients calculated by removing the outlier values of bMIND. corr(${\mathcal{L}}_{1}$,GPdiff) denotes the correlation coefficient between ${\mathcal{L}}_{1}$ scores and GPdiff among the three complete methods. The three correlation scores are placed in the first row of each dataset and are not tied to DeconRNASeq

Two observations are salient. First, all ${\mathcal{L}}_{2}$ scores are considerably higher than all ${\mathcal{L}}_{1}$ scores, indicating superiority of ${GP}_{ref}$ to $Q$ in capturing the distribution of expression patterns. Second, for ${\mathcal{L}}_{1}$ scores incomplete methods are generally superior to two complete methods. However, deconf_original is a complete method but has the best ${\mathcal{L}}_{1}$ in three datasets (50,0), (3,0), (3,0.7), and NMF is a complete method but has the best ${\mathcal{L}}_{1}$ in one dataset (50,0.7). The six incomplete methods have similar ${\mathcal{L}}_{1}$ scores which are lower than the best complete method but higher than the remaining two complete methods.

Since the log-likelihood scores were jointly determined by both mixture coefficients and signature matrices (or distributions), it may be misleading to directly correlate the log-likelihood scores with each aspect of decomposition. It is more sensible to correlate the ${\mathcal{L}}_{1}$ (and ${\mathcal{L}}_{2}$) scores with the differences of mixture coefficient vectors among the six incomplete methods, and with the differences of $P(x|$ $\gamma ,\pi )$ terms among the three complete methods, since the incomplete methods have identical $P(x|$ $\gamma ,\pi )$ terms. ${\mathcal{L}}_{1}$ scores are strongly anti-correlated with $P(x|$ $\gamma ,\pi )$ errors among complete methods. In contrast, correlations of the log likelihood scores and the mixture coefficient vector errors among incomplete methods are less clear. By removing the outlier values of bMIND, ${\mathcal{L}}_{2}$ scores are strongly anti-correlated with the mixture coefficient vector errors. Yet ${\mathcal{L}}_{1}$ scores are positively correlated with the mixture coefficient vector errors in the two datasets with $\delta =0.7$, indicating that ${\mathcal{L}}_{1}$ scores are sensitive to the additive noise.

Supplementary file 1: Figure S1A-B displays the mixture coefficients of the ground truth and six incomplete methods in two virtual bulk datasets ((50,0), (3,0.7)). In both datasets, DWLS has among the top three ${\mathcal{L}}_{1}$ scores and the lowest deviation from the true mixture coefficients. Supplementary file 1: S1C-D displays the signature matrices of three complete methods in the same virtual datasets. In both datasets, deconf_original has the highest ${\mathcal{L}}_{1}$ scores and the lowest deviation from the true $P(x|$ $\gamma ,\pi )$ distribution among the complete deconvolution methods.

### Mouse bulk-level and single-cell RNASeq data

Beyond in-silico mixtures, we also assessed how backward deconvolution gauged the performance of deconvolution algorithms on true mouse bulk-level data. We downloaded and processed the mouse bulk-level and single-cell RNASeq data from Tabula Muris Senis [46], selected 4439 marker genes, 164 bulk samples from four tissue types (fat, heart, limb, and liver), and 33,777 cells from nine cell types (endothelial cells, fibroblast/mesenchymal cells, epithelial cells, immune cells, smooth muscle cells, skeletal muscle cells, endocardial cells, Schwann cells, and hepatocyte cells). The procedure of selecting and processing the data is reported in Sect. "Selecting marker genes and cells from four single-cell and bulk-level datasets".

Table 2 reports the log-likelihood scores for nine deconvolution algorithms on the subset of the mouse sc-RNASeq data. Similar to the in-silico mixtures, each incomplete method has a superior ${\mathcal{L}}_{2}$ than ${\mathcal{L}}_{1}$. However, unlike in-silico mixtures the six incomplete methods yield superior ${\mathcal{L}}_{1}$ scores than most of three complete deconvolution algorithms (with one exception that NMF (a complete method) is superior to lsfit (an incomplete method)). Scaden, DWLS and RADs are the best in terms of both ${\mathcal{L}}_{1}$ and ${\mathcal{L}}_{2}$ scores.

**Table 2:** Inference results on a true bulk-level mouse RNASeq dataset. celltypeaccuracy1 and celltypeaccuracy2 denote the accuracy rates of cell type assignments in single-cell RNASeq data in terms of the $P(x|$ $\gamma ,\pi )$ models derived from $Q$ and $GP_{ref}$ respectively

Algorithm	${\mathcal{L}}_{1}$	${\mathcal{L}}_{2}$	Celltypeaccuracy1	Celltypeaccuracy2	Corr(${\mathcal{L}}_{1}$,celltypeaccuracy1)	Corr(${\mathcal{L}}_{2}$,celltypeaccuracy2)
DeconRNASeq	− 126,652,924	− 70,722,794	0.159334	0.446563	0.81920	0.935071
lsfit	− 127,418,320	− 75,076,458	0.144117	0.231097		
DWLS	− 124,996,556	− 68,933,318	0.345136	0.830008		
bMIND	− 126,171,077	− 71,343,524	0.224643	0.450234		
RADs	− 125,051,051	− 68,943,491	0.345491	0.826988		
Scaden	− 124,988,528	− 68,889,855	0.347090	0.828883		
NMF	− 127,306,451		0.053171			
deconf_original	− 132,632,460		0.004914			
deconf_fast	− 132,569,233		0.086151			

The correlation coefficients between ${\mathcal{L}}_{1}$ and celltypeaccuracy1 and between ${\mathcal{L}}_{2}$ and celltypeaccuracy2 are also reported in the two last columns

**Fig. 2:**
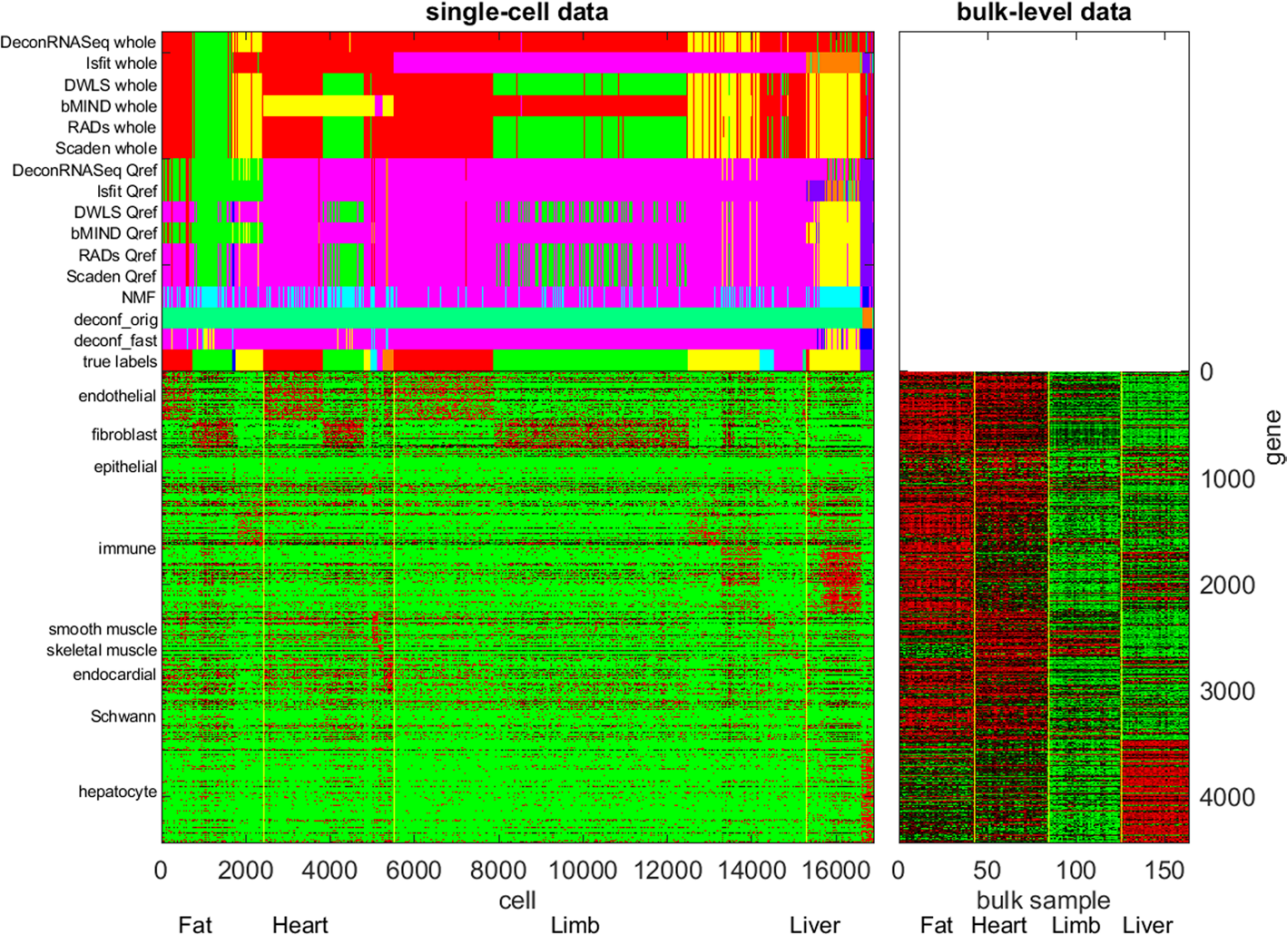
**A** Single-cell RNASeq data from selected tissue types in the Tabula Muris database, and true and predicted cell types inferred from multiple deconvolution algorithms. The lower portion visualizes the sorted sc-RNASeq data of four tissues and nine cell types. Genes (rows) are sorted by the marker gene groups of each cell type. Cells (columns) are sorted by first tissue types and second cell types. Yellow vertical lines mark the boundaries of cells belonging to each tissue. Red and green colors indicate high and low expressions. For instance, fat tissues comprise endothelial, fibroblast and immune cells, which have elevated expressions in the marker genes of the corresponding groups. The upper portion visualizes the true and predicted cell types derived from each deconvolution algorithm. The bottom row indicates the true cell types of the sorted cells. Red: endothelial cells, green: fibroblast cells, blue: epithelial cells, yellow: immune cells, cyan: smooth muscle cells, magenta: skeletal muscle cells, orange: endocardial cells, green–blue: Schwann cells, purple: hepatocyte. The remaining rows indicate the predicted cell types from each deconvolution algorithm. For six incomplete methods (DeconRNASEq, lsfit, DWLS, bMIND, RADs, Scaden), we report the predicted cell types according to to ${\mathcal{L}}_{1}$ ($Q_{ref}$) and ${\mathcal{L}}_{2}$ (whole). **B** Bulk-level RNASeq data of the same tissue types. Genes (rows) follow the same order as Fig. 2A, and samples (columns) are sorted by the four tissue types

The sample mixture coefficients in the true bulk-level data are unknown. Instead of comparing inferred mixture coefficients with ground truth, we predicted cell types in the test sc-RNASeq data according to each model (Eq. 6) and calculated their accuracy rates. Intriguingly, the accuracy rates for both $Q$ and ${GP}_{ref}$ models are highly correlated with ${\mathcal{L}}_{1}$ and ${\mathcal{L}}_{2}$. Moreover, Scaden, DWLS and RADs possess the highest ${\mathcal{L}}_{1}$ and ${\mathcal{L}}_{2}$ as well as the best accuracy rates for both $Q$ and ${GP}_{ref}$ models. Figure 2 visualizes the bulk-level and single-cell data and the true and predicted cell types from each model. The ${GP}_{ref}$ models of Scaden, DWLS and RADs offer near 83% accurate predictions on cell types, indicating that these three methods are indeed the best algorithms in this dataset.

The strong correlations between the log-likelihood scores and various error metrics inspired us to examine the relations of these quantities in a semi-formal way. Assume the sc-RNASeq data were generated by the aforementioned model. Denote ${\theta }^{*}\equiv ({P}_{{\theta }^{*}}\left(\pi |s\right),{P}_{{\theta }^{*}}\left(x|\gamma ,\pi \right))$ the true parameter values of the model, and $\theta \equiv ({P}_{\theta }\left(\pi |s\right),{P}_{\theta }\left(x|\gamma ,\pi \right))$ the undetermined parameter values which are formulated as random variables in a Bayesian framework. Assume an infinite amount of data were generated from ${\theta }^{*}$, then the likelihood score asymptotically approximates the KL divergence between ${\theta }^{*}$ and $\theta$:

9 $$\mathcal{L}({\varvec{x}};\theta )\to {E}_{x\sim P\left({\varvec{x}}|{\theta }^{*}\right)}[\text{log}P({\varvec{x}}|\theta )]=-{D}_{KL}\left({\theta }^{*}\parallel \theta \right).$$

We then derive the approximation error of the bulk-level data deconvolution. Equation 1 gives the true deconvolution outcomes. Suppose $\widehat{Q}$ and $\widehat{P}$ are the approximated signature and mixture coefficients matrices respectively, then the reconstructed bulk-level gene expression data becomes:

10 $$\widehat{E}=\widehat{Q}\cdot \widehat{P}.$$

Each bulk-level expression vector is the average of a collection of single-cell expression vectors sampled from $P\left({\varvec{x}}|{\theta }^{*}\right)$. If $P\left({\varvec{x}}|{\theta }^{*}\right)$ is given, then $Q$ is obtained from the mean sampled from $P(x|\gamma ,\pi )$, and $P$ is $P(\pi |s)$. Hence $Q\cdot P$ is the mean of the expression data sampled from $P\left({\varvec{x}}|{\theta }^{*}\right)$. The approximation error then becomes the variance of the expression values.

11 $$E_{{x\sim P\left( {{\varvec{x}}{|}\theta^{*} } \right)}} [x - E_{{x\sim P\left( {{\varvec{x}}{|}\theta^{*} } \right)}} x]^{2} \equiv var_{{x\sim P\left( {{\varvec{x}}{|}\theta^{*} } \right)}} x.$$

If the true distribution $P\left({\varvec{x}}|{\theta }^{*}\right)$ is not given, but instead the parameters $\theta$ are estimated from finite data, then the approximation error becomes:

12 $$|E - \hat{Q} \cdot \hat{P}|^{2} \equiv E_{{x\sim P\left( {{\varvec{x}}{|}\theta^{*} } \right)}} [x - E_{{x\sim P\left( {{\varvec{x}}{|}\theta } \right)}} x]^{2} = var_{{x\sim P\left( {{\varvec{x}}{|}\theta^{*} } \right)}} x + (E_{{x\sim P\left( {{\varvec{x}}{|}\theta^{*} } \right)}} x - E_{{x\sim P\left( {{\varvec{x}}{|}\theta } \right)}} x)^{2} .$$

The first term indicates the variance from the true distribution, and the second term indicates the bias between the true and estimated distributions.

Finally, the errors on $\widehat{Q}$ and $\widehat{P}$ are approximated as:

13 $$|{Q}^{*}-\widehat{Q}{|}^{2}\propto |{P}_{\theta }\left(x|\gamma ,\pi \right)-{P}_{{\theta }^{*}}\left(x|\gamma ,\pi \right){|}^{2}.$$

14 $$|{P}^{*}-\widehat{P}{|}^{2}\propto |{P}_{\theta }\left(\pi |s\right)-{P}_{{\theta }^{*}}\left(\pi |s\right){|}^{2}.$$

For incomplete methods, in a relaxed condition ${P}_{{\theta }^{*}}\left(x|\gamma ,\pi \right)$ is given from the reference signature matrix or distribution. There is a close relation between the KL divergence (Eq. 9) and square error (Eq. 14) of the mixture coefficients pertaining to $\theta$ and ${\theta }^{*}$. For complete methods such as NMF, $-{D}_{KL}\left({\theta }^{*}\parallel \theta \right)$ has the composite effect of both $Q$ and $P$. Hence the relation between the KL divergence and each of the approximation error terms (Eqs. 13 and 14) is less obvious.

### Bulk-level and single-cell brain RNASeq data of autism spectrum disorder (ASD) subjects

The two datasets in Sects. "In-silico mixture of mouse sc-RNASeq data"-"Mouse bulk-level and single-cell RNASeq data" are considered as simple problems for deconvolution as the cells and bulk samples from the selected tissue types possess quite distinct gene expression patterns among the selected marker genes. To justify the utility of backward deconvolution in more challenging scenarios, we downloaded and processed bulk-level and single-cell RNASeq data of brains of distinct regions from ASD patients and normal subjects. This is a more challenging dataset as the differences of cell type composition and gene expression signatures are much subtler between samples of different brain regions or diagnosis than between samples of different organs. Here we employed backward deconvolution to demonstrate that human brains exhibited different cell type compositions between ASD patients and normal controls as well as between distinct brain regions.

**Fig. 3:**
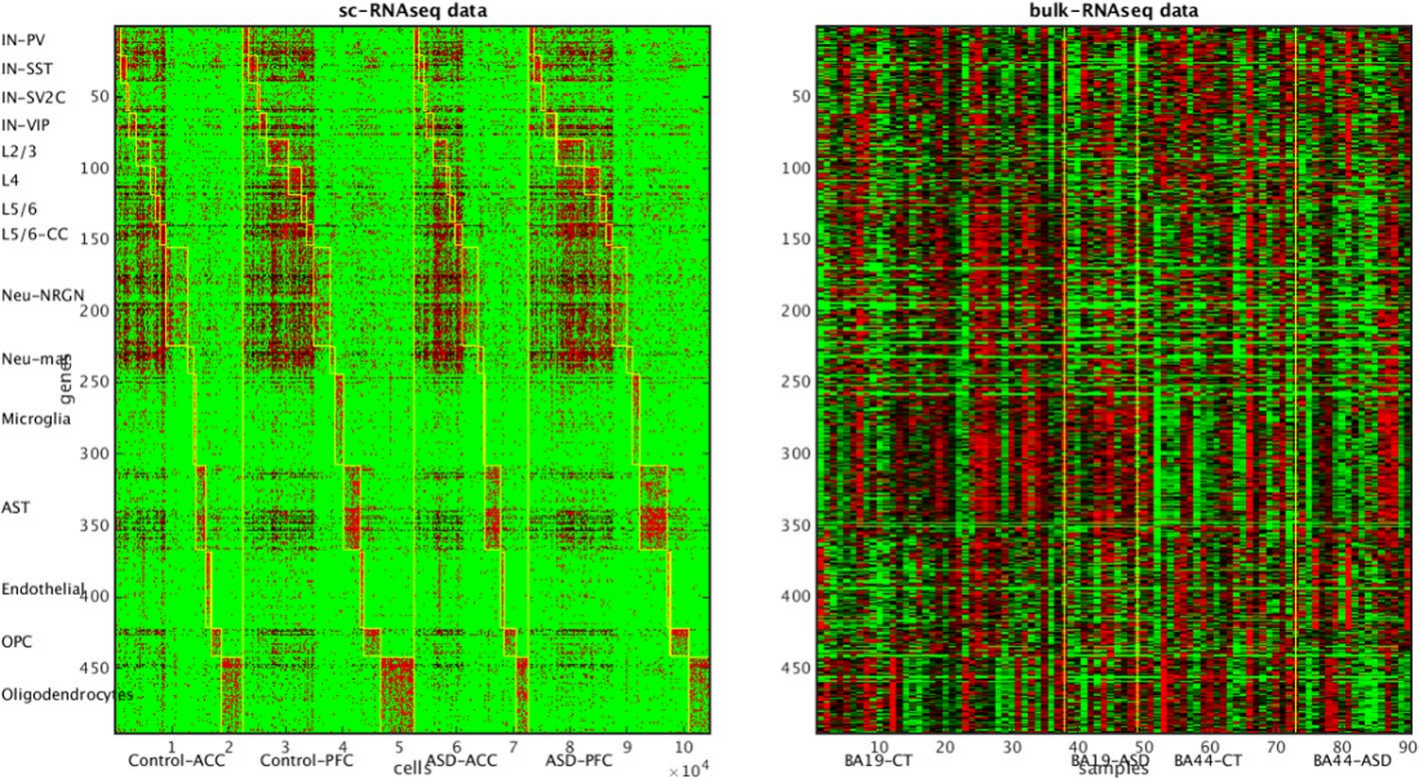
**A** Single-cell RNASeq data of 104,559 cells from 15 cell types and the samples of two brain regions (ACC and PFC) of ASD patients and normal controls. Cells (columns) are sorted by first sample subtypes and second cell types. Marker genes (rows) are sorted by the 15 gene groups. **B** Bulk-level RNASeq data of 90 samples from two brain regions (BA19 and BA44) of ASD patients and normal controls. BA19 and BA44 are matched to ACC and PFC according to the ${\mathcal{L}}_{2}$ scores of the three best deconvolution methods (DWLS, Scaden and RADs) linking bulk-level and single-cell data. The genes (rows) follow the same order as in Panel A

Figure 3 displays the ASD data of 500 selected marker genes. Cells belonging to the four sample subtypes (control-ACC, control-PFC, ASD-ACC, ASD-PFC) comprise 15 cell types including various types of neuron cells and supporting cells such as microglia and astrocytes. The four sample subtypes possess different cell type compositions in sc-RNASeq data by visual inspection (Fig. 3A), yet these differences can be attributed to biases in sampling cells from the prepared tissues. The bulk-level data look even less informative than the sc-RNASeq data (Fig. 3B). To answer the aforementioned question (whether brains possess different cell type compositions between phenotypes and/or brain regions), we applied the backward deconvolution framework to the dataset and compared the inferred mixture coefficients of the top-ranking forward deconvolution methods.

Table 3A reports the log-likelihood scores of nine deconvolution algorithms. Similar to the results on other datasets, the incomplete methods have superior likelihood scores than the complete methods, ${\mathcal{L}}_{2}$ scores are superior to ${\mathcal{L}}_{1}$ scores, and the cell type prediction accuracy rates are highly correlated with the log likelihood scores. The three best methods according to both ${\mathcal{L}}_{2}$ and cell type prediction accuracy rates are Scaden, RAD, and DWLS.

**Table 3:** (A) Log likelihood scores and cell type prediction accuracy rates of nine deconvolution methods on ASD data. (B) The mixture coefficients of 15 cell types and 4 sample subtypes inferred from the nine deconvolution methods

Algorithm	${\mathcal{L}}_{1}$	${\mathcal{L}}_{2}$	Celltypeaccuracy1	Celltypeaccuracy2	Corr (${\mathcal{L}}_{1}$,celltypeaccuracy1)	Corr (${\mathcal{L}}_{2}$,celltypeaccuracy2)
DeconRNASeq	− 54,150,640	− 24,919,550	0.104765	0.200559	0.66388599	0.94326443
lsfit	− 61,029,630	− 27,158,730	0.057136	0.125175		
DWLS	− 51,213,420	− 23,443,550	0.148186	0.47258		
RADs	− 51,435,480	− 22,810,060	0.148587	0.587215		
Scaden	− 51,230,630	− 22,786,240	0.148339	0.577746		
bMIND	− 51,183,850	− 24,953,340	0.147038	0.361732		
NMF	− 37,583,340		0.065189			
deconf_original	− 70,571,330		0.03116			
deconf_fast	− 87,230,560		0.011286			

Method	Type	AST	Endo	IN- PV	IN- SST	IN-SV2C	IN-VIP	L2/3	L4	L5/6	L5/6-CC	Neu-NRGN	Neu-mat	Microglia	OPC	Oligo
DeconRNASeq	C- ACC	0.815014	0.014504	0	0.01383	0	0	0.015032	0	0	0	0.119822	0	0.018534	0	0.003263
	C-PFC	0.790456	0.009751	0	0.015746	0	0	0.014589	0	0	0	0.150608	0	0.018849	0	0
	A-ACC	0.802913	0.028238	0	0.028456	0	0	0.012852	0	0	0	0.072208	0	0.05371	0	0.001624
	A-PFC	0.795489	0.030014	0	0.005591	0.00001	0	0.032963	0	0	0	0.120227	0	0.015705	0	0
lsfit	C-ACC	0.721664	0.003745	0	0.002524	0	0	0	0	0	0	0.243547	0	0.02852	0	0
	C-PFC	0.660301	0.000435	0	0.006695	0	0	0	0	0	0	0.293568	0	0.039002	0	0
	A-ACC	0.733702	0.013471	0	0.009925	0	0	0	0	0	0	0.145046	0	0.097855	0	0
	A-PFC	0.733582	0.011534	0	0.002321	0	0	0	0	0	0	0.228535	0	0.024028	0	0
DWLS	C-ACC	0.179895	0.013783	0	0.000405	0	0	0.000103	0.000065	0	0	0.71	0	0.022375	0.000521	0.072852
	C-PFC	0.13114	0.00847	0	0.000593	0	0.000002	0.000028	0.000475	0	0	0.779866	0	0.010976	0.00052	0.067932
	A-ACC	0.273308	0.026986	0	0.000724	0	0	0	0	0	0.000936	0.5618	0	0.035759	0.001122	0.099365
	A-PFC	0.288821	0.021803	0	0.000346	0	0	0.000827	0.000315	0	0.000181	0.57733	0.00008	0.030363	0.002283	0.07765
RADs	C-ACC	0.106409	0.027622	0.208448	0.046872	0.057068	0.058741	0.056206	0.006363	0.024275	0.00816	0.088008	0.20521	0.103671	0.001898	0.001049
	C-PFC	0.052355	0.006836	0.249805	0.042912	0.113257	0.052575	0.05985	0.005709	0.011034	0.006847	0.07094	0.235246	0.091727	0.000048	0.000859
	A-ACC	0.09508	0.041666	0.12897	0.072873	0.094812	0.05279	0.080129	0.040333	0.017442	0.029661	0.055346	0.1749	0.111473	0.003858	0.000667
	A-PFC	0.162366	0.03581	0.070282	0.036871	0.049754	0.037279	0.025404	0.036071	0.005126	0.01073	0.032493	0.423555	0.07293	0.000812	0.000516
Scaden	C-ACC	0.022203	0.028944	0.011365	0.025337	0.014786	0.009444	0.005893	0.014843	0.005505	0.041375	0.706187	0.054895	0.018086	0.009988	0.031148
	C-PFC	0.015554	0.017987	0.009019	0.021521	0.011126	0.00677	0.004457	0.012432	0.00367	0.037438	0.769491	0.052389	0.011956	0.006501	0.019691
	A-ACC	0.041947	0.046077	0.013946	0.030137	0.018808	0.013095	0.00813	0.018107	0.008073	0.04504	0.600263	0.0587	0.025291	0.014752	0.057636
	A-PFC	0.041723	0.041666	0.016212	0.033849	0.02139	0.015221	0.008791	0.020241	0.008461	0.046294	0.591661	0.063212	0.026428	0.016374	0.048479
bMIND	C-ACC	0.111416	0.158586	0	0	0.000128	0.000605	0	0	0	0	0.445061	0.002886	0.089885	0.018248	0.173186
	C-PFC	0.104695	0.144144	0	0	0	0.003587	0	0	0	0	0.476865	0.007821	0.079346	0.008566	0.167965
	A-ACC	0.127472	0.172887	0	0	0	0.000455	0	0	0	0	0.403032	0	0.089938	0.008566	0.197650
	A-PFC	0.139144	0.153769	0	0	0	0	0	0	0	0	0.418148	0.002211	0.095092	0.011828	0.179809
NMF	C-ACC	0.087197	0.047483	0.097451	0.118518	0.073153	0.034896	0.075423	0.027707	0.030044	0.121345	0.067621	0.037579	0.084124	0.072323	0.072323
	C-PFC	0.068533	0.01988	0.072038	0.145517	0.146789	0.023775	0.085688	0.022573	0.031537	0.085336	0.093431	0.01387	0.0942	0.064532	0.032302
	A-ACC	0.087121	0.062626	0.057712	0.06126	0.050598	0.040762	0.083917	0.06255	0.090612	0.079831	0.079418	0.052539	0.075183	0.056186	0.059686
	A-PFC	0.073709	0.051048	0.030009	0.054107	0.149215	0.023257	0.075055	0.066388	0.047288	0.188118	0.050244	0.055665	0.084819	0.027251	0.023828
deconf_original	C-ACC	0.011378	0	0.242557	0.066413	0.02569	0	0.114999	0.174324	0.015784	0	0.228939	0	0.119914	0	0
	C-PFC	0.002212	0	0.245898	0.064838	0.070024	0	0.108441	0.208252	0.005945	0	0.197003	0	0.097387	0	0
	A-ACC	0.024455	0	0.154608	0.041209	0.089988	0	0.103626	0.224922	0.060959	0	0.223108	0	0.077126	0	0
	A-PFC	0.029864	0	0.328292	0.017151	0.016027	0	0.049799	0.197055	0.053059	0	0.226404	0	0.082348	0	0
deconf_fast	C-ACC	0	0.105727	0.040319	0	0	0.003684	0	0.512854	0	0	0.323284	0	0	0.000783	0.01335
	C-PFC	0	0.100382	0.044801	0	0	0	0	0.537188	0	0	0.30954	0	0	0	0.00809
	A-ACC	0	0.171907	0.040279	0	0	0.005953	0	0.431024	0	0	0.348746	0	0	0	0.002091
	A-PFC	0	0.058996	0.131925	0.000954	0	0.014966	0	0.531976	0	0	0.255023	0	0	0	0.00616

The abbreviations of sample subtypes are: C-CC: Control-ACC, C-PFC: Control-PFC, A-ACC: ASD-ACC, A-PFC: ASD_PFC. The abbreviations of cell types are: AST: astrocytes, Endo: endothelial cells, Neu-NRGN: NRGN-expressing cells, Oligo: oligodendrocytes

We then examined the inferred $P(\pi |s)$ tables of the three best methods (Table 3B). Intriguingly, for two of the top three methods (Scaden and DWLS) ASD samples have higher fractions of astrocytes than control samples conditioned on the brain regions. In other words, $P\left(\pi =\text{astrocyte}|s=\text{ASD}, \text{ACC}\right)>P\left(\pi =\text{astrocyte}|s=\text{control},\text{ ACC}\right)$, and $P\left(\pi =\text{astrocyte}|s=\text{ASD}, \text{PFC}\right)>P\left(\pi =\text{astrocyte}|s=\text{control}, \text{PFC}\right)$. In addition, ASD samples have lower fractions of NRGN-neurons than control samples conditioned on both brain regions. Finally, ACC samples have higher fractions of oligodendrocytes than PFC samples conditioned on the phenotypes (ASD or control).

### Breast cancer bulk-level and single-cell RNASeq data

**Fig. 4:**
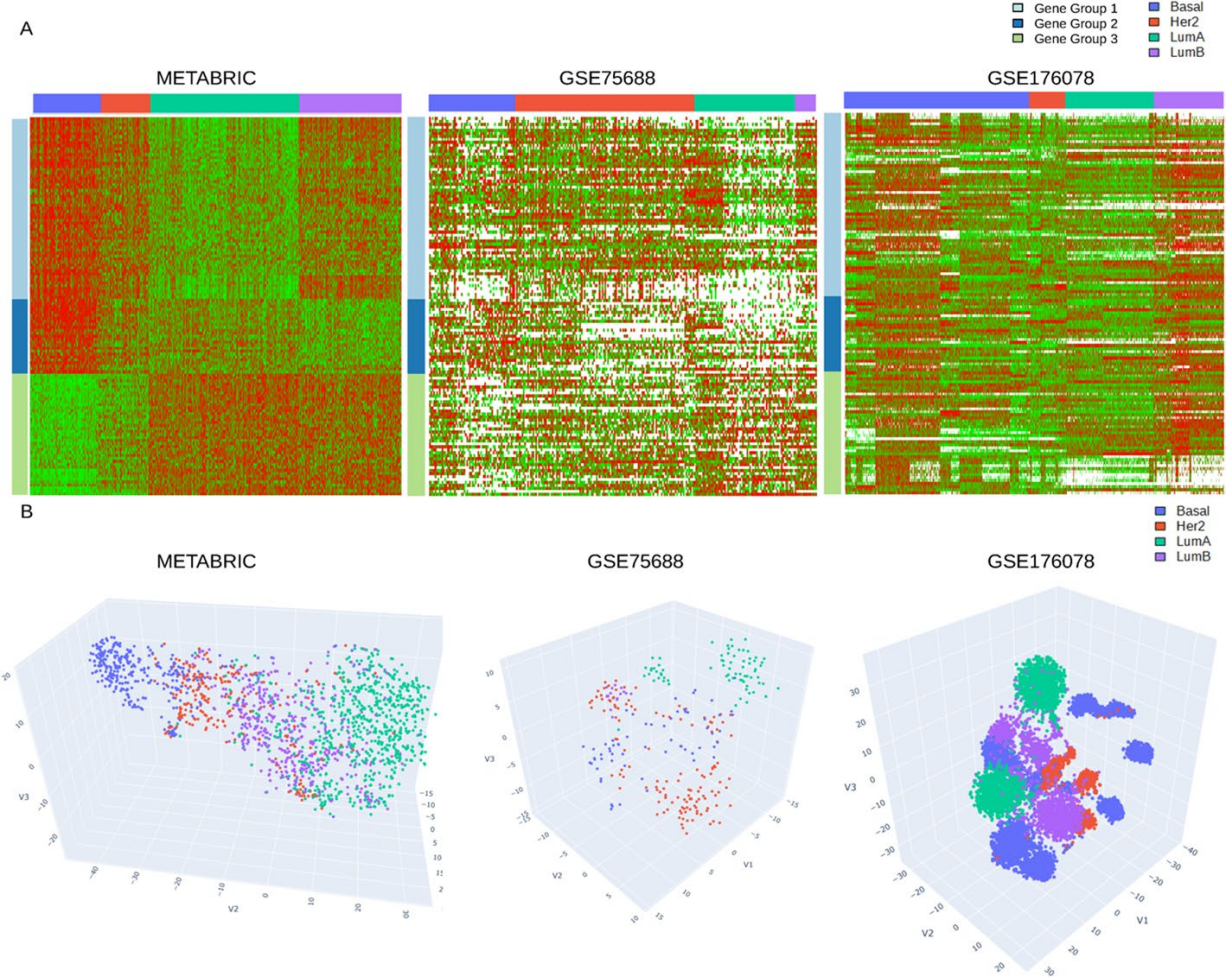
**A** Expressions of marker genes on one bulk-level (METABRIC) and two single-cell (GSE75688 and GSE176078) breast cancer datasets. Genes (rows) are sorted by three marker gene groups, and tumors/cells (columns) are sorted by the four breast cancer subtypes. Red and green colors indicate high and low expressions, and white color indicates missing entries. **B** Their corresponding t-SNE 3D projections of the data in Fig. 3A. Each point represents the expression data of one tumor/cell (a column in Fig. 3A). The colors of points indicate their breast cancer subtypes

Breast cancers are classified into four subtypes [54]: basal, Her2-enriched, luminal A and luminal B. We expanded the well-known PAM50 genes [49] to 127 genes and categorized them into three gene groups (Sect. "Selecting marker genes and cells from four single-cell and bulk-level datasets" and [50]). The bulk-level samples of the four subtypes possess distinct combinatorial expression patterns on the three gene groups (Fig. 4A, left panel). However, it is unclear whether the combinatorial expression pattern of each PAM50 subtype is dominated by one cell type or attributed to a mixture of several common cell types.

**Fig. 5:**
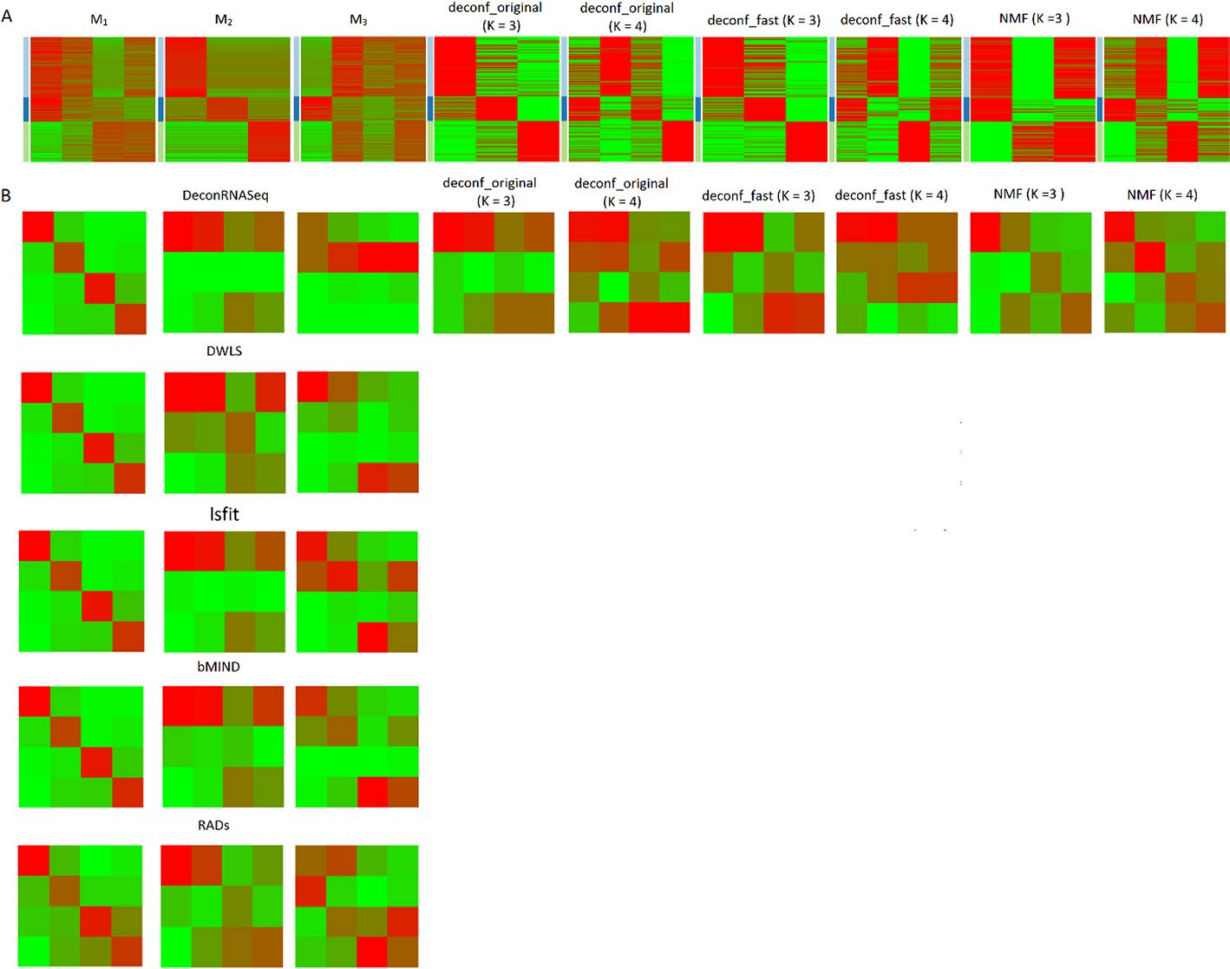
**A** Signature matrices and **B** Conditional probability matrices $P(\pi |s)$ generated for breast cancer data. In each panel of (A), each row indicates a gene, and each column indicates an inferred cell type. Genes are sorted by their marker gene groups as in Fig. 4. The first three panels display the signature matrices of the reference models $M_{1} - M_{3}$ manually constructed from the bulk-level data. The remaining panels display the signature matrices inferred from three complete deconvolution algorithms with $K = 3$ and $K = 4$. In each panel of **B**, each row indicates an inferred cell type, and each column indicates a breast cancer subtype. For five incomplete algorithms (DeconRNASeq, DWLS, lsfit, bMIND, RADs), we applied the three reference signature matrices $M_{1} - M_{3}$ to infer the mixture coefficients and derived the $P(\pi |s)$ matrices. The results are displayed in $3 \times 3$ panels. For three complete algorithms (deconf_original, deconf_fast, NMF), we set $K = 3$ and $K = 4$ and displayed the inferred $P(\pi |s)$ matrices in $1 \times 6$ panels

To answer this question, we employed backward deconvolution to one bulk-level breast cancer RNASeq dataset (METABRIC, [55]) and two single-cell RNASeq datasets (GSE75688, [56]; GSE176078, [57]), and focused on cancer cells in the two sc-RNASeq datasets. We termed GSE75688 and GSE176078 small and large datasets as they comprised 281 and 12,019 cancer cells respectively after processing (Sect. "Selecting marker genes and cells from four single-cell and bulk-level datasets"). For each incomplete method, we proposed three simple hypotheses about the expression patterns of the cell types underlying the PAM50 subtypes. Hypothesis 1 (${M}_{1}$) stipulates that the tumors of each subtype are dominated by one cell type (Fig. 5A, panel 1). ${M}_{2}$ stipulates that tumors of each subtype are mixtures of three cancer cell types which have elevated expressions in one gene group each (Fig. 5A, panel 2). ${M}_{3}$ serves as a negative control of ${M}_{1}$ by rearranging the sample subtypes and gene groups from the bulk-level data to maximize the difference from ${M}_{1}$ (Fig. 5A, panel 3). We checked whether certain models consistently outperformed other models in the two sc-RNASeq datasets.

Figure 4A displays marker gene expressions on three datasets. Combinatorial expression patterns of PAM50 subtypes are salient in METABRIC and deteriorated in single-cell datasets. Figure 4B displays the t-SNE projections of samples in the three datasets. METABRIC samples of the four subtypes are clearly separated, but cells in the two single-cell datasets are clustered primarily by patient identities (annotations not shown) rather than PAM50 subtypes.

Table 4 reports the 21 ${\mathcal{L}}_{1}$ scores and 15 ${\mathcal{L}}_{2}$ scores on breast cancer data, and Fig. 5 visualizes the signature matrices and inferred mixture coefficients of the bulk-level data. Similar to the mouse data, each incomplete method has a superior ${\mathcal{L}}_{2}$ than ${\mathcal{L}}_{1}$ in all three datasets. BIC scores on bulk-level data serve as sanity check as the true model of the expression patterns of sample subtypes (${M}_{1}$) is given. Indeed, for each incomplete method both ${\mathcal{L}}_{1}$ and ${\mathcal{L}}_{2}$ scores follow the order $M_{1} > M_{2} > M_{3}$, and for two complete methods the model of $K = 4$ outperforms the model of $K = 3$, but their scores are inferior to those of incomplete methods. A complete method NMF is the only anomaly, as it has the ${\mathcal{L}}_{1}$ score comparable to the ${\mathcal{L}}_{1}$ scores of the best incomplete methods, and the model of $K = 3$ is superior to the model of $K = 4$.

**Table 4:** Inference results on breast cancer RNASeq dataset

Algorithm	Bulk ${\mathcal{L}}_{1}$	Bulk ${\mathcal{L}}_{2}$	Small sc ${\mathcal{L}}_{1}$	Small sc ${\mathcal{L}}_{2}$	Large sc ${\mathcal{L}}_{1}$	Large sc ${\mathcal{L}}_{2}$
DeconRNASeq $M_{1}$	− 581,501	− 487,260	− 59,963	− 53,452	− 1,930,048	− 1,694,837
DeconRNASeq $M_{2}$	− 619,231	− 504,911	− 68,157	− 54,586	− 2,146,903	− 1,712,380
DeconRNASeq $M_{3}$	− 622,655	− 525,474	− 65,928	− 54,398	− 2,119,167	− 1,726,796
lsfit $M_{1}$	− 581,512	− 487,250	− 59,967	− 53,451	− 1,930,248	− 1,694,759
lsfit $M_{2}$	− 611,947	− 504,469	− 66,077	− 54,440	− 2,107,018	− 1,706,659
lsfit $M_{3}$	− 620,176	− 521,745	− 65,889	− 53,995	− 2,111,609	− 1,713,069
DWLS $M_{1}$	− 581,532	− 487,250	− 59,970	− 53,451	− 1,930,361	− 1,694,823
DWLS $M_{2}$	− 610,417	− 504,692	− 65,585	− 54,417	− 2,092,453	− 1,706,134
DWLS $M_{3}$	− 620,539	− 521,886	− 65,974	− 54,007	− 2,114,633	− 1,715,287
bMIND $M_{1}$	− 581,606	− 487,253	− 59,976	− 53,445	− 1,930,604	− 1,694,717
bMIND $M_{2}$	− 611,036	− 504,584	− 65,838	− 54,488	− 2,097,675	− 1,705,636
bMIND $M_{3}$	− 620,246	− 522,259	− 65,931	− 54,108	− 2,113,414	− 1,719,336
RADs $M_{1}$	− 581,752	− 487,734	− 59,863	− 53,389	− 1,926,330	− 1,692,877
RADs $M_{2}$	− 610,387	− 504,721	− 65,683	− 54,453	− 2,091,412	− 1,705,257
RADs $M_{3}$	− 621,098	− 521,355	− 66,002	− 53,726	− 2,111,351	− 1,701,333
NMF $S_{3}$	− 595,958		− 61,424		− 1,970,872	
NMF $S_{4}$	− 605,731		− 63,171		− 2,016,043	
deconf_original $S_{3}$	− 631,875		− 65,098		− 2,088,872	
deconf_original $S_{4}$	− 616,722		− 64,338		− 2,044,264	
deconf_fast $S_{3}$	− 620,474		− 63,701		− 2,052,652	
deconf_fast $S_{4}$	− 612,236		− 63,228		− 2,024,205	

The regularized ${\mathcal{L}}_{1}$ and ${\mathcal{L}}_{2}$ scores of 21 models on the bulk-level dataset and two single-cell datasets are reported

The ${\mathcal{L}}_{1}$ scores on sc-RNASeq datasets are largely congruent with expectation. $M_{1}$ has superior scores than $M_{2}$ and $M_{3}$ for all incomplete methods, and complete methods are generally inferior to the $M_{1}$ scores of incomplete methods (except NMF). The ${\mathcal{L}}_{2}$ scores on sc-RNASeq datasets are also compatible with expectation. On the small sc-RNASeq data, the ${\mathcal{L}}_{2}$ scores of each incomplete method follow the order $M_{1} > M_{3} > M_{2}$. On the large sc-RNASeq data, they follow the order $M_{1} > M_{2} > M_{3}$.

**Fig. 6:**
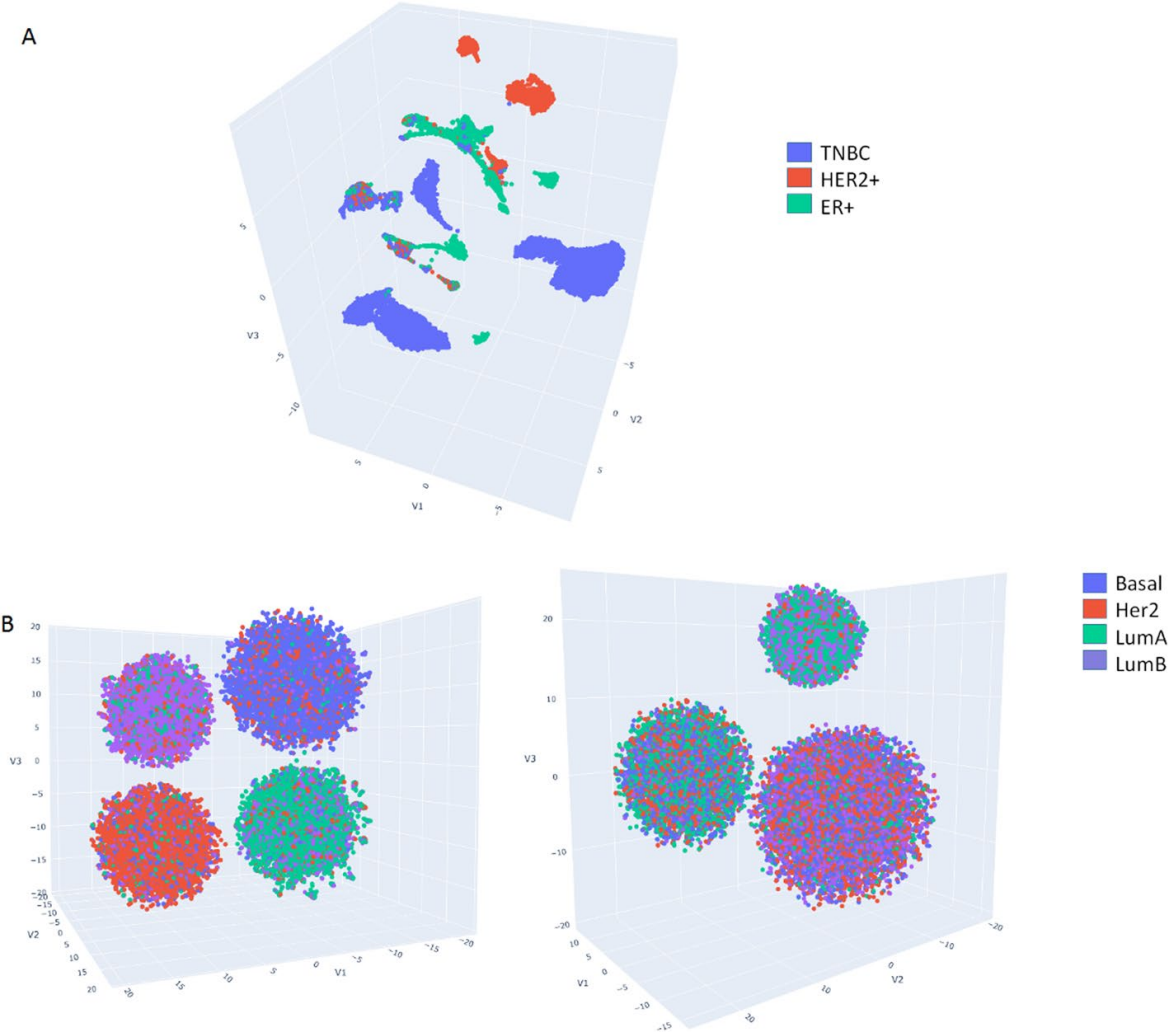
(**A**) 3D t-SNE projection of the breast cancer sc-RNASeq data from [57]. Cells are colored by the subtypes of the samples they belong to. TNBC corresponds to the basal-like subtype, and ER + corresponds to luminal A and B subtypes. **B** 3D t-SNE projections of the breast cancer virtual cells simulated from the two models $M_{1}$ and $M_{2}$ respectively. Cell clusters correspond to the hidden cell types, and cell colors denote the PAM50 subtypes of their samples

The superior log likelihood scores of $M_{1}$ offer indirect evidence supporting the strength of $M_{1}$ to fit the data. To provide direct evidence supporting the strength of $M_{1}$, we found another independent breast cancer sc-RNASeq dataset [57], clustered the cells and annotated their PAM50 subtypes, and then compared the clustering outcomes with those of two virtual sc-RNASeq datasets simulated from $M_{1}$ and $M_{2}$. Figure 6 visualizes the clustering outcomes of the real breast cancer sc-RNASeq data (Fig. 6A) and those of the two virtual datasets simulated from $M_{1}$ and $M_{2}$ (Fig. 6B). Supplementary file 5: Table S2 reports the confusion tables of clustering outcomes of the real breast cancer sc-RNASeq data (Table S2A) and those of the $M_{1}$ and $M_{2}$ simulated data (Table S2B and S2C). Intriguingly, the $M_{1}$ data resembles the real data more closely than the $M_{2}$ data. In the real sc-RNASeq data, cells are clustered primarily by their PAM50 subtypes. This clustering pattern is nearly reproduced in the $M_{1}$ data. In contrast, in the $M_{2}$ data cells are clustered by the three hidden cell types rather than their PAM50 labels. Consequently, the model $M_{1}$ better describes the breast cancer sc-RNASeq data than an alternative model $M_{2}$.

### Low-grade glioma bulk-level and single-cell RNASeq data

Low-grade glioma (LGG) patients in The Cancer Genome Atlas (TCGA) data were classified into three subtypes according to the mutation states of Idh1 gene and chromosome 1p/19q co-deletion [58]: Idh1 mutation with or without co-deletion and wild type. We identified 61 marker genes and labeled them to three gene groups enriched with neuron development, cell cycle, and immune response, respectively.

Supplementary file 2: Figure S2A displays expressions of the marker genes on LGG bulk-level [58] and single-cell [59] datasets, and Supplementary file 2: Figure S2B displays the t-SNE projections of samples in the two datasets. Similar to the breast cancer data, it is difficult to discern the underlying cell types from the gene expression visualization and t-SNE projections alone.

We considered the same three hypotheses $M_{1} - M_{3}$ and checked which hypothesis better fit the sc-RNASeq data. Supplementary file 6: Table S3 reports the 18 ${\mathcal{L}}_{1}$ scores and 15 ${\mathcal{L}}_{2}$ scores on one bulk-level and one single-cell LGG data, and Supplementary file 3: Figure S3 visualizes the signature matrices and inferred mixture coefficients of the bulk-level data. Similar to Sects. "In-silico mixture of mouse sc-RNASeq data"-"Breast cancer bulk-level and single-cell RNASeq data", the ${\mathcal{L}}_{2}$ scores are higher than the ${\mathcal{L}}_{1}$ scores of all models in both datasets. $M_{2}$ has the best ${\mathcal{L}}_{1}$ for each incomplete method, and the three complete methods have superior ${\mathcal{L}}_{1}$ than incomplete methods. In contrast, in the bulk-level data the ${\mathcal{L}}_{2}$ scores of incomplete methods follow the order $M_{1} > M_{2} > M_{3}$. In the single-cell data the ${\mathcal{L}}_{2}$ scores of incomplete methods follow the order $M_{1} > M_{3} > M_{2}$. Consequently, the $M_{1}$ models derived from $GP_{ref}$ yield the best BIC scores.

## Discussion

We propose a backward deconvolution framework to infer cell type gene expression signatures and compositions by integrating both bulk-level and single-cell RNASeq data. It has several unique benefits. First, it compares and selects a decomposition model from multiple candidates rather than sticking to one particular decovolution algorithm and/or hypothesis. Second, it handles the sc-RNASeq data with high-level noise, abundant zero entries, and no cell type annotations by constructing the reference signature matrix and distribution from bulk-level data with stronger hypotheses. Third, the log-likelihood scores provide a common metric for the joint effect of signature matrices (or distributions) and mixture coefficients in fitting the sc-RNASeq data. Fourth, the log-likelihood scores can be evaluated without knowing bulk sample mixture coefficients or single-cell annotations, hence can be applied in a wider range of datasets.

Several important discoveries are drawn from the analysis of five datasets. First, there is no universally superior deconvolution algorithm over all datasets, as each dataset has its best performing algorithm. Nevertheless, overall three incomplete deconvolution algorithms—DWLS, RADs and Scaden—tend to be superior to other algorithms in most datasets. Second, in the mouse data where the single cell annotations and/or bulk sample mixtures are provided, the log-likelihood scores of nine deconvolution methods are largely compatible with the deviations of mixture coefficients, gene expression conditional probabilities, or cell type assignments from the ground truth. Third, in the human brain data ASD samples tend to possess higher fractions of astrocytes and lower fractions of NRGN-expressing neurons than control samples. The first observation was reported in the study of the ASD sc-RNASeq data [47], and both observations were manifested in both bulk-level and single-cell data. Fourth, in the cancer data with no single-cell annotations and abundant zero entries, the model that tumors of each subtype are dominated by one cell type ($M_{1}$) outperforms an alternative model that each cell type possesses elevated expressions on one gene group and low expressions on the remaining gene groups ($M_{2}$). Moreover, in an independent breast cancer sc-RNASeq dataset, cells were clustered primarily by their sample subtypes (PAM50 subtypes). By comparing with the sc-RNASeq data simulated by the two hypothetical models, we found that the clustering patterns of the real data resembled $M_{1}$ the most. The results are not definitive since we have not tested $M_{1}$ against many alternative models. Nevertheless, superiority of $M_{1}$ to $M_{2}$ has supporting evidence from prior studies. Tumors of the four breast cancer subtypes have similar expression profiles as the cell types in normal breast epithelium. It is thus widely hypothesized that the four breast cancer subtypes may arise from distinct normal cell types [60] or mutations or genetic rearrangements occurring in different populations of stem cells and progenitor cells [61]. Tumors of the three LGG subtypes are likely derived from the subclones arising from Idh1 mutations and chromosome 1p/19q co-deletion events [62]. Therefore, cancer cells of a tumor subtype likely inherit the expression signatures from their tissues of origin or founder cells, and are relatively homogeneous. Heterogeneity is present primarily in the interactions between cancer cells and different types of normal cells such as multiple families of immune cells, stromal cells, fibroblasts, and others [63]. By contrast, even though a tumor may comprise multiple subclones, cancer cells of these subclones are likely derived from the same cell type. Thus the cancer cells from the same tumor subtypes may share the common expression patterns on the marker genes. This postulation by no means claims that cancer cells are homogeneous. Rather, we think homogeneity/heterogeneity is relative to the examined features (gene expressions). Expressions of cancer cells from multiple subclones are likely heterogeneous in the genes involved in the molecular alterations segregating these subclones (sequence mutations, copy number variations, structural variations, etc.), but homogeneous among the marker genes selected from bulk-level data analysis. Fifth, all the ${\mathcal{L}}_{2}$ scores are superior to all the ${\mathcal{L}}_{1}$ scores, and ${\mathcal{L}}_{2}$ often better matches anticipation than ${\mathcal{L}}_{1}$. This suggests that $Q$ is less reliable to estimate $P(x|\gamma ,\pi )$ compared to $GP_{ref}$. $Q$ collapses the entries of each gene in the cells of each type into one number by taking an average, but $GP_{ref}$ retains the entries of all the cells of each type. Hence the latter estimates $P(x|\gamma ,\pi )$ from far more entries than the former and is more accurate.

The analysis of each dataset possesses some customized procedures. Most of these procedures pertain to selection of marker genes, gene groups, sample subtypes and cell types in the data. These procedures facilitate deconvolution operations and make the results more interpretable, but are strictly speaking not part of the backward deconvolution framework. These variables are treated as given in the framework. Users interested in applying the backward deconvolution programs into their data can ignore our customized procedures and directly provide sample subtypes, cell types, and gene group labels of their data.

Several open problems remain in the present study. When sc-RNASeq data have poor quality or no annotations, the models underlying signature matrices and distributions are manually constructed from the bulk-level data. Manual construction is preferable currently as we aim to compare a few simple and interpretable hypotheses about cancer cell type heterogeneity. However, in the long run it is desirable to have an algorithm capable of generating simple and sensible hypotheses for backward deconvolution. Although the log-likelihood scores combine the joint effects of mixture coefficients and cell type specific gene expression patterns, the downside is that these two effects are entangled. Better statistical methods are required to disentangle the contributions of the two factors. In the current formulation of marginal likelihood function (Eq. 3), the effect of $$P(x|$$ $$\gamma ,\pi )$$ often outweighs that of $$P(\pi |$$ $$s)$$ because in each cell the former term multiplies over all marker genes yet the latter term appears only once. Hence differences in estimated mixture coefficients are likely overwhelmed by estimated cell type specific gene expression distributions. Similar problems arise in topic models of natural language processing, and several techniques have been proposed to correct the asymmetric contributions [64, 65]. We plan to adopt some of these methods in the future development of backward deconvolution. Albeit we proposed a probabilistic graphical model in generating the sc-RNASeq data, we did not adopt a fully Bayesian approach to evaluate the likelihood scores. Instead of integrating over the possible conditional probability $$P\left( {\pi {|}s} \right)$$ and $$P(x|$$ $$\gamma ,\pi )$$ values, we estimated their values from the bulk data deconvolution outcomes and plugged the estimated values into the likelihood function. A fully Bayesian approach is conceivable if we introduce proper prior distributions of $$P\left( {\pi {|}s} \right)$$ and $$P(x|$$ $$\gamma ,\pi )$$ and employ standard Bayesian inference methods to evaluate the marginal likelihood scores over both cell type values and parameter values.

### Supplementary Information

Supplementary Material 1. Figure S1: (A) Mixture coefficients of the ground truth (top panel) and those inferred from six incomplete deconvolution methods for the artificial mixture data with η, δ = (50,0), (B) Mixture coefficients for the artificial mixture data with η, δ = (3,0.7), (C) Signature matrices inferred from three complete deconvolution methods for the artificial mixture data with η, δ = (50,0), (D) Signature matrices for the artificial mixture data with η, δ = (3,0.7).

Supplementary Material 2. Figure S2: (A) Visualization of gene expression LGG data on the bulk level (TCGA) and single-cell level (GSE151506), (B) t-SNE visualization of the same two expression datasets.

Supplementary Material 3. Figure S3: Deconvolution results on LGG data. (A) Signature matrices of three hypothetical models (M_1_--M_2_.) for incomplete deconvolution methods and those inferred from three complete deconvolution methods, (B)Mixture coefficients inferred from three complete deconvolution methods (deconf_original, deconf_fast, and NMF) and five incomplete deconvolution methods.

Supplementary Material 4. Table S1: The numbers of grids with enriched or depleted zero entries in six datasets.

Supplementary Material 5. Table S2: (A) The confusion table of the clustering outcomes on the breast cancer sc-RNASeq dataset GSE161529. Rows indicate clusters of cells, and three columns indicate the PAM50 subtypes of the samples encompassing the cells, (B) The confusion table of the virtual data simulated by M_1_, rows and columns correspond to cell types (π) and sample subtypes (s) respectively, (C) The confusion table of the virtual data simulated by M1.

Supplementary Material 6. Table S3: The log-likelihood scores 18 probabilistic graphical models on LGG bulk-level and single-cell RNASeq data.

## Data Availability

All data generated or analyzed during this study are included in this published article and its supplementary information files.

## Availability of source codes

We have implemented the backward deconvolution algorithm in R, and deposited the source codes and their description on github.com/chyeang/backward-deconvolution/.
