# Supplementary figures and images for: Assessing transcriptomic heterogeneity of single-cell RNASeq data by bulk-level gene expression data
Source: BMC Bioinformatics. 2024 Jun 12;25:209. doi: 10.1186/s12859-024-05825-3 (PMC11167951; doi:10.1186/s12859-024-05825-3)

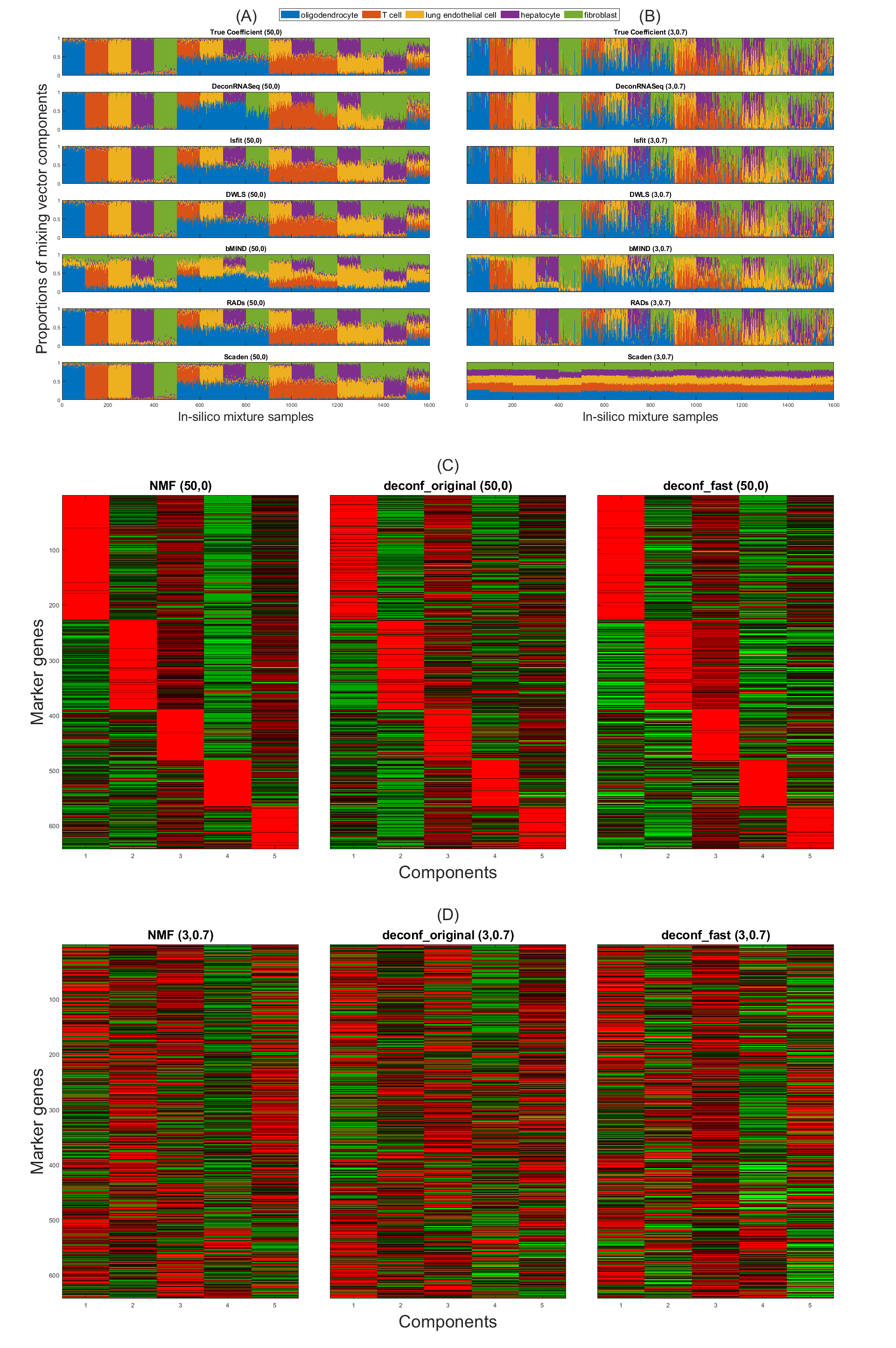

Supplement: Supplementary file 1 — Supplementary Material 1. Figure S1: (A) Mixture coefficients of the ground truth (top panel) and those inferred from six incomplete deconvolution methods for the artificial mixture data with η, δ = (50,0), (B) Mixture coefficients for the artificial mixture data with η, δ = (3,0.7), (C) Signature matrices inferred from three complete deconvolution methods for the artificial mixture data with η, δ = (50,0), (D) Signature matrices for the artificial mixture data with η, δ = (3,0.7). [file 12859_2024_5825_MOESM1_ESM.tiff]

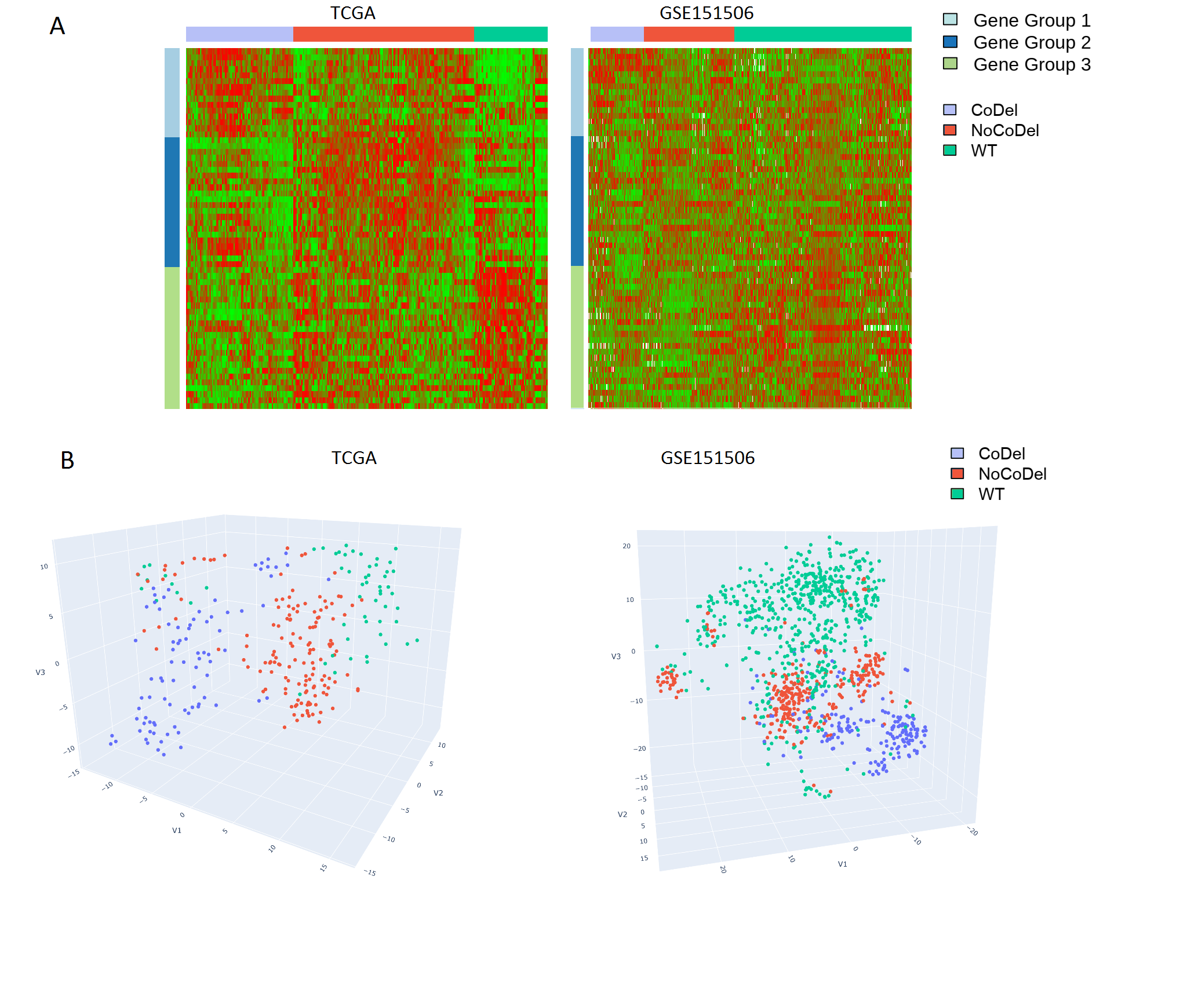

Supplement: Supplementary file 2 — Supplementary Material 2. Figure S2: (A) Visualization of gene expression LGG data on the bulk level (TCGA) and single-cell level (GSE151506), (B) t-SNE visualization of the same two expression datasets. [file 12859_2024_5825_MOESM2_ESM.tiff]

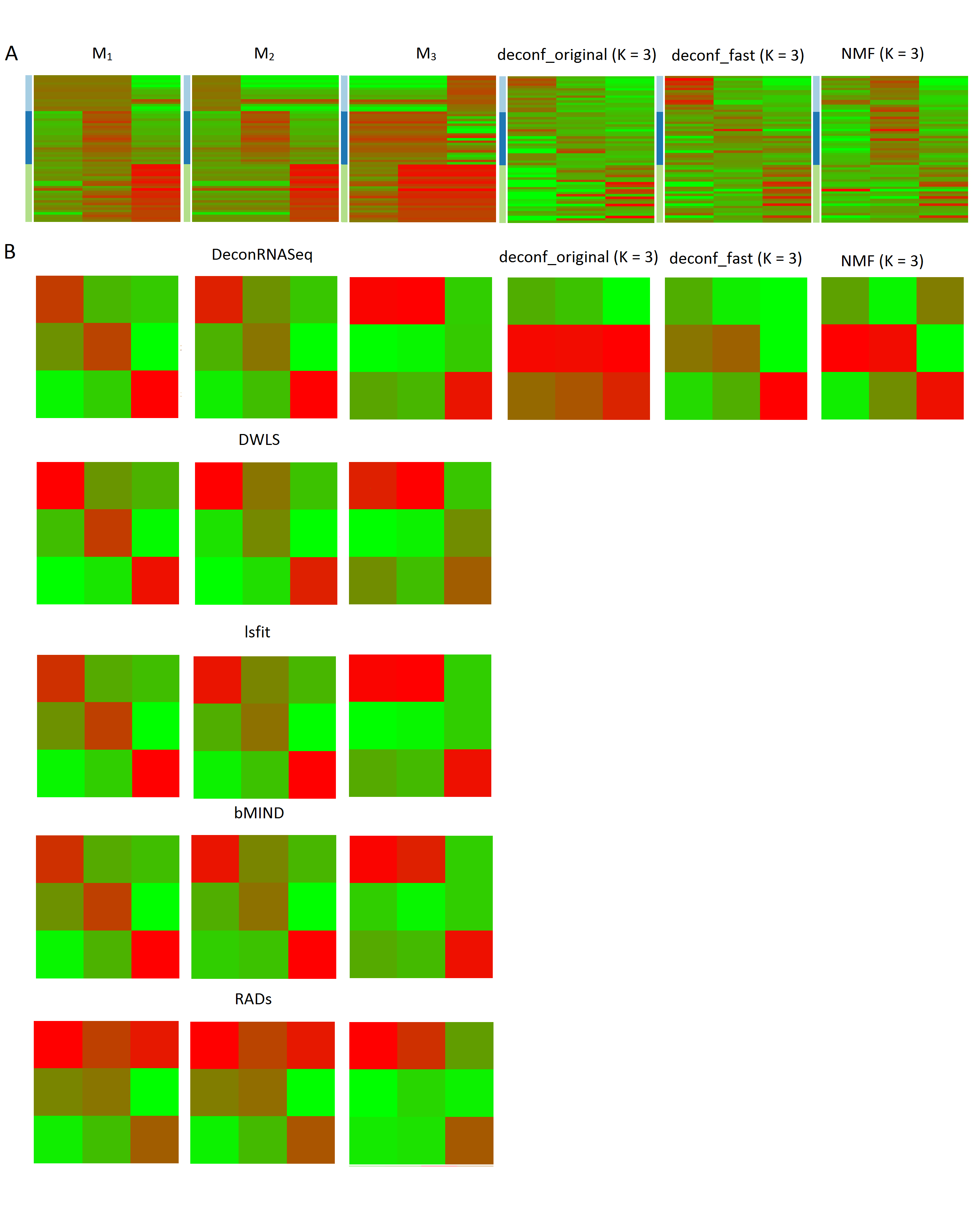

Supplement: Supplementary file 3 — Supplementary Material 3. Figure S3: Deconvolution results on LGG data. (A) Signature matrices of three hypothetical models (M1--M2.) for incomplete deconvolution methods and those inferred from three complete deconvolution methods, (B)Mixture coefficients inferred from three complete deconvolution methods (deconf_original, deconf_fast, and NMF) and five incomplete deconvolution methods. [file 12859_2024_5825_MOESM3_ESM.tif]
